# Evolutionary analysis of angiosperm dehydrin gene family reveals three orthologues groups associated to specific protein domains

**DOI:** 10.1038/s41598-021-03066-5

**Published:** 2021-12-13

**Authors:** Alejandra E. Melgar, Alicia M. Zelada

**Affiliations:** 1 Laboratorio de Agrobiotecnología, Departamento de Fisiología, Biología Molecular y Celular, Facultad de Ciencias Exactas y Naturales, Universidad de Buenos Aires, Buenos Aires, Argentina; 2 Instituto de Biodiversidad y Biología Experimental y Aplicada, Consejo Nacional de Investigaciones Científicas y Técnicas-Universidad de Buenos Aires (IBBEA, CONICET-UBA), Buenos Aires, Argentina

**Keywords:** Evolution, Plant sciences

## Abstract

Dehydrins (DHNs) are a family of plant proteins that play important roles on abiotic stress tolerance and seed development. They are classified into five structural subgroups: K-, SK-, YK-, YSK-, and KS-DHNs, according to the presence of conserved motifs named K-, Y- and S- segments. We carried out a comparative structural and phylogenetic analysis of these proteins, focusing on the less-studied KS-type DHNs. A search for conserved motifs in DHNs from 56 plant genomes revealed that KS-DHNs possess a unique and highly conserved N-terminal, 15-residue amino acid motif, not previously described. This novel motif, that we named H-segment, is present in DHNs of angiosperms, gymnosperms and lycophytes, suggesting that HKS-DHNs were present in the first vascular plants. Phylogenetic and microsynteny analyses indicate that the five structural subgroups of angiosperm DHNs can be assigned to three groups of orthologue genes, characterized by the presence of the H-, F- or Y- segments. Importantly, the hydrophilin character of DHNs correlate with the phylogenetic origin of the DHNs rather than to the traditional structural subgroups. We propose that angiosperm DHNs can be ultimately subdivided into three orthologous groups, a phylogenetic framework that should help future studies on the evolution and function of this protein family.

## Introduction

Plants have to deal with different environmental stresses that can negatively affect their growth and development. Loss of intracelullar water in response to abiotic stresses like drought, salinity and low temperature results in the accummulation of Late Embryogenesis Abundant (LEA) proteins in different vegetative tissues. LEA proteins belong to a large group of proteins known as "hydrophilins" characterized by glycine-rich, highly hydrophilic disordered amino acid sequences^1^. Based on sequence similarity, LEAs are classified into 7 Pfam families distinguished by the presence of different conserved motifs that are named LEA1, LEA2, LEA3, LEA4, LEA5, SMP and dehydrins (DHNs), the last one also known as group II or D11 LEA proteins^2,3^.

Dehydrins (DHNs) constitute a biochemically and evolutionarily distinct group of LEAs with a highly modular structure consisting of a combination of different conserved motifs, variable in number and type, interspersed within weakly conserved amino acid segments. The presence of at least one conserved lysine rich-motif, named the K-segment, is usually used as a condition to define a protein as a dehydrin^4^. Two other conserved motifs have been described, the Y- and S-segments, that in conjuction with the K-segment are the basis for the general classification of DHNs into 5 structural subgroups: KnS, SKn, YnK, YnSKn and Kn-DHNs, where n refers to the number of repetitions of a given motif^5^.

The Y-segment is usually located at the N-terminus of the protein in one or several tandem copies, while the S-segment is always found in one copy per protein^5^. Recently, Strimbeck^6^ described a new conserved motif present in a subgroup of SK-DHNs, named the F-segment. These conserved motifs are surrounded by less conserved sequences denoted Phi-segments, with a higher Gly, Thr, and Glu content^7^.

DHNs are involved in abiotic stress tolerance, acting in the protection of membranes, cryoprotection of enzymes, interaction with DNA and protection against reactive oxygen species^8–12^. Usually, the biochemical and functional characteristics of these proteins are analysed within the framework of conserved structural domains, without regard for the evolution of the family. The phylogenetic relationships of DHNs have been studied in many different plants, but most of these studies are limited to one genus or species^13–15^. Only recently, a comprehensive understanding of the evolutionary history of DHNs has been attempted. A phylogenetic and structural analysis of a large number of plant DHNs by Riley et al.^16^ suggests that the ancestral DHN belonged to a Kn or SKn group, and that YSKn and YKn-DHNs first arose in angiosperms^16^. Artur^17^ showed that angiosperm DHNs with Y- and F- segments belong to two different orthologue groups that can be distinguished by synteny conservation^17^. The evolutionary origin of KS-DHNs, on the other hand, is still elusive.

Here, we present a thorough phylogenetic and structural analysis of DHNs obtained from a wide spectrum of plant genomes. Even though KS-DHNs have previously been described only in a handful of species, we show that this DHN group is actually present in all angiosperms as well as in gymnosperms and lycophytes, indicating an ancient origin. We show that KS-dehydrin genes share a conserved synteny neighbourhood in angiosperm genomes and possess a conserved N-terminal domain, that we named H-segment, and propose that all angiosperm DHNs belong to one of three orthologue groups, the H, F and Y groups.

## Methods

### Dehydrin protein sequences database construction and MEME search motifs

Initially, DHN proteins were obtained by searching plant genomes or transcriptomes with the Hidden Markov Model (HMM) profile assigned to the DHN protein family (PF00257), downloaded from the Pfam database (http://pfam.xfam.org/), using the HMMER 3.1 software (http://hmmer.org/). The HMM profile was used to search the Phytozome v13 database (https://phytozome-next.jgi.doe.gov/) which harbours 56 genomes from species spanning the whole viridiplantae clade, including one rodophyte, nine chlorophytes, two bryophytes (*Ceratodon purpureus* and *Physcomitrella patens*), the lychophyte *Selaginella moellendorffii*, the angiosperm species *Amborella trichopoda* and *Nymphaea colorata* and a subset of 9 monocots and 28 eudicots representing different families. To include gymnosperm species in our search, we employed the Gymno PLAZA 1.0 database^18^ and the ConGeniE database (http://congenie.org/) which contain the transcriptomes of *Ginkgo biloba, Picea abies* and *Picea glauca.*

As a first step to identify the conserved motif structures of DHN proteins, we used the MEME software (http://meme-suite.org/)^19^ with the following parameters: number of motifs = 8, motif width = 6 to 20, and number of sites for each motif = 2 to 1000. Since we noticed that KS-type DHNs were underrepresented in this preliminary DHN database, we searched the National Center for Biotechnology Information (NCBI) database to retrieve homologues of *Arabidopsis thaliana* HIRD11 using the Blastp algorithm, and the new KS-DHN sequences were used to construct a HMM profile specific for this DHN group. In parallel, HMM profiles were also constructed for F- and Y-DHNs. Finally, the three HMM profiles were used to reanalyse the databases and an unbiased database was constructed that is presented in Supplementary Data S1.

The conserved motif structures of DHN proteins in the unbiased database were identified using MEME software to find recurrent ungapped motifs assuming that each sequence may contain any number of non-overlapping motifs. The results presented correspond to an analysis made with the following parameters: number of motifs = 8, motif width = 6 to 16, and number of sites for each motif = 2 to 1000 (Supplementary Fig. S1). The E-values of the different motifs predicted by MEME for our DHN database were compared to E-values calculated from the same sequenced randomly shuffled using the same MEME run parameters to confirm the significance of the discovered motifs.

### Multiple sequence alignments and phylogenetic tree construction

Multiple sequence alignment analyses of DHN protein sequences from different structural subgroups were performed using Clustal Omega^20^ and T-coffee^21^ with default parameters, and visualized using Jalview^22^. Those that displayed better alignments of different conserved motifs are presented as Supplementary information (Supplementary Figs. S2–S10).

The phylogenetic tree was constructed using an MSA obtained with Clustal Omega^20^ that included only angiosperm DHNs, in order to prevent very divergent sequences from reducing the quality of the alignment. Protein sequences from the unbiased database were chosen so as to represent all angiosperm families and keeping a similar number of species per family. Species with DHNs with atypical structures like *Salix purpurea* and *Populus trichocharpa* were not included. Phylogenetic trees were estimated by the Maximum Likelihood (ML) method as implemented in IQ-TREE (http://iqtree.cibiv.univie.ac.at/)^23^. The best-fit model was determined as being JTT + F + R4 by ModelFinder^24^ with free rate heterogeneity. A total of one thousand bootstrap samplings were run using Ultrafast Boostrap aproximation^25^. The resulting tree was visualized using iTOL^26^.

Evolutionary relationships of bryophyte dehydrins were estimated with the Maximum Likelihood (ML) method as implemented in the NGPhylogeny website (https://ngphylogeny.fr/)^27^ using PhyML 3.0^28^ with the LG amino acid substitution model^29^ and the GAMMA model with invariant sites for rate heterogeneity.

### Microsynteny analysis

For microsynteny analysis of selected DHN genes, the corresponding proteins were identified in the NCBI database by pairwise BLASTP searches. Annotations with 100% identity were selected and the genomic context analysed using the NCBI Genome Data Viewer (GDV). Protein sequences of ten to twenty genes flanking both sides of DHN genes were compared between species, using the loci of *A. trichopoda* DHN genes as references. Reciprocal BLASTP analysis were used to confirm homology, with sequences that matched with an E-value of < 10 − 5 being considered homologous to each other.

### Calculation of physicochemical properties and disorder prediction of DHNs proteins

The theoretical physicochemical properties of DHNs such as grand average hydropathicity index (GRAVY), molecular weight (MW), isoelectric point (pI) and glycine percentage were calculated with the ProtParam tool of Expasy (https://web.expasy.org/protparam/). The GRAVY index indicates the hydrophobicity of the protein and was calculated as the sum of the hydropathy values (Kyte and Doolittle parameters) of all amino acids divided by the sequence length. Proteins with positive GRAVY scores are hydrophobic whereas proteins with negative GRAVY scores are hydrophilic. The fold index of proteins was estimated using the FoldIndex© software (https://fold.weizmann.ac.il/fldbin/findex). Disorder tendencies of DHNs were analysed using IUPred2A^30^ (https://iupred2a.elte.hu/), long disorder (default option) was used to predict global structural disorder.

## Results

### Unbiased genome-wide identification of dehydrins in Viridiplantae genomes

To understand the evolution of KS-DHNs and their relationship to the other structural subgroups (YnSKn-, YnKn- SKn- and Kn-DHNs), we performed a genome-wide searches to identify the repertoires of DHN genes across 56 genomes of Viridiplantae species, including members of chlorophytes and streptophytes. The initial screening was made using a Hidden Markov Model (HMM) profile defined for dehydrin family proteins (Pfam00257)^31^. Surprisingly, when we analysed the sequences retrieved, we noticed that well known KS-DHNs, such as the HIRD11 dehydrin from *Arabidopsis thaliana* (At1g54410)^32^ and the ZmDHN13 from *Zea mays*^8^ were not detected by the algorithm. That prompted us to hypothesize that a Pfam00257 is not sensitive enough to recognize KS-DHNs. To overcome this limitation we built three different HMM profiles: one (KS-HMM) using KS-DHN sequences from angiosperm genomes identified by Blastp searches using *A. thaliana* HIRD11, and two other profiles (F-HMM and Y-HMM) based on angiosperm proteins belonging to the F- and Y-DHNs orthologous groups recently described^17^.

After searching with Pfam00257 and the group-specific HMM profiles, we recovered a total of 305 non-redundant sequences from genomes of representative species of bryophytes (4), lycophytes (1), gymnosperms (3) and angiosperms (36) (Supplementary Data S1). No sequences were retrieved from algal genomes, suggesting that DHNs might have emerged in land plants^7^.

**Figure 1:**
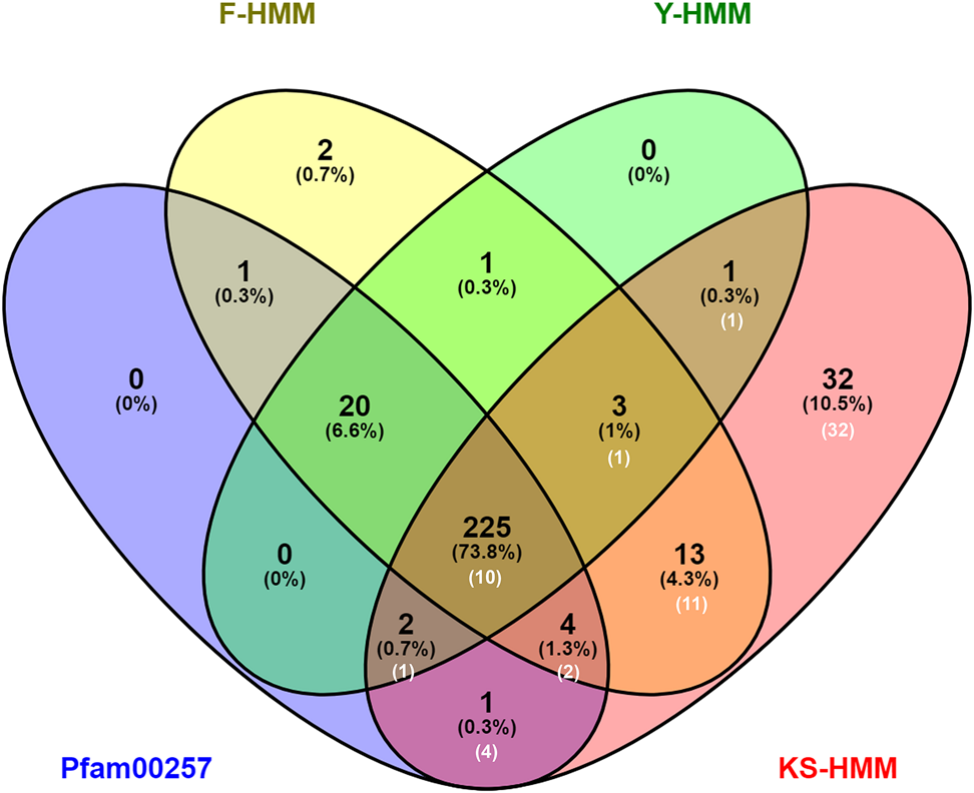
DHNs identified in Viridiplantae genomes by different HMM profiles. Sequences retrieved from species genomes using F-HMM, Y-HMM, KS-HMM profiles constructed in this study and Pfam00257 the Pfam profile for dehydrin family proteins are displayed as a Venn diagram. White numbers indicate the number of KS-DHNs present in each subset. Note that most KS-DHNs are not recovered using Pfam00257.

Remarkably, the KS-HMM profile displayed an increased sensitivity in recognising DHN homologues, since it was able to identify 92.2% of DHN proteins, while the Pfam00257-based HMM identified 83% (Fig. 1). From a total of 62 DHNs exhibiting the KS-architecture, only 17 could be retrieved using the Pfam00257 profile, confirming its poor performance in recognizing KS-DHNs. While F-HMM has a better perfomance and could recognize 24 KS-DHNs, only the KS-HMM profile was able to retrieve all KS-DHNs. Indeed, 32 KS-DHNs could only be retrieved using the KS-HMM profile. Conversely, the KS-HMM profile failed to recognize 24 DHN sequences that were identified by the other HMM profiles. No DHNs were retrieved solely by Pfam00257, indicating that group specific-HMM profiles are necessary for a thorough search of DHN proteins in genomes.

### Analysis of conserved protein motif and classification of the dehydrin database

**Figure 2:**
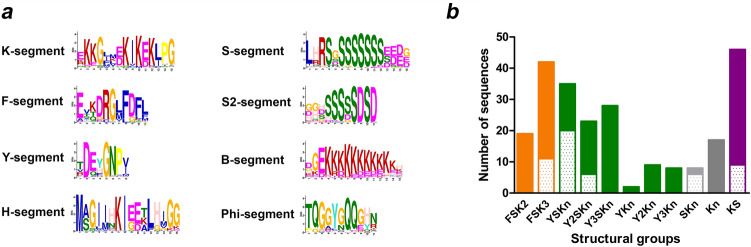
Identification of conserved protein motifs and structural classification of DHNs. (**a**) LOGO representation of the different conserved motifs detected by MEME in the set of DHNs of the unbiased database. (**b**) Number of members of each angiosperm DHN structural subgroup identified in the unbiased database. We distinguished FSK2 and FSK3 structural subgroups in accordance to Strimbeck^6^. All classified DHNs belonging to 19 angiosperm families are listed in Supplementary Data S1. The dotted pattern indicates monocots, while the filled pattern indicates eudicots.

We used MEME to check for the presence of known dehydrin motifs (K- Y- F- and S-segments) and to discover putative novel motifs (Supplementary Fig. S1). The conserved motifs and the number of DHNs in the structural subgroups detected are shown in Fig. 2.

**Figure 3:**
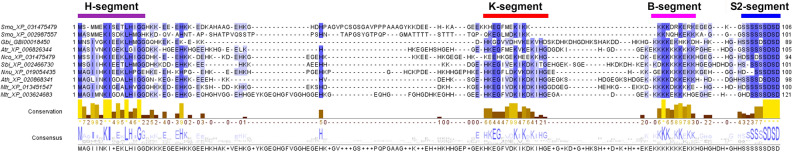
Conservation of KS-DHNs in vascular plants. Multiple sequence alignment of KS-DHNs (HKS-DHNs) of representative angiosperms (*A. trichopoda*, *S. bicolor*, *N. nucifera*, *A. thaliana*, *M. truncatula*) and non-angiosperms (the lycophyte *S. moellendorffii* and the gymnosperm *G. biloba*) performed with T-Coffee and visualised with Jalview. The H-, K-, B and S-segments are indicated. Note that the general structure of the proteins is conserved in all vascular plant groups.

We confirmed the presence of the K-segment in 302 of the 307 dehydrins identified by homology searches based on HMM profiles. MEME failed to recognize a K-segment in a few proteins, all of which from non-angiosperm species. However, these proteins all possess a degenerate, less conserved K-segment, as well as other DHN motifs, indicating that they are bona fide DHNs. This is the case, for instance, for DHNs from the lycophyte *S. moellendorffii* and the gymnosperm *Ginkgo biloba* (Fig. 3).

We identified 75 DHNs bearing a unique F-segment located in the N-terminal region of the proteins, that we classified within the FSKn-DHN structural subgroup. The F-segment predicted with our database is similar to the one described by Strimbeck^6^ (Fig. 2a). Importantly, even a search with a specific F-HMM profile failed to identify FSKn-DHNs in the genomes of four bryophytes and one lycophyte, but we did find them in the three gymnosperms included in this study, *Picea abies*, *Picea glauca* and *G. biloba*, confirming that this group arose in seed plants. We observed an expansion of the FSKn-DHN group in the Pinaceae clade, in accordance with previous observations^33^, but only three DNHs were identified in *G. biloba*, two with a F-segment (FSK2 and FK2) and a third being a KS-DHN (see below). In angiosperms, the FSKn-DHN subgroup is mostly comprised of FSK2 and FSK3 proteins (Fig. 2b) but, interestingly, only FSK3-DHNs are found in monocots and in the early divergent eudicot *Nelumbo nucifera*, as well as in the basal angiosperms *A. trichopoda* and *N. colorata* (Supplementary Figs. S2–S3), suggesting that FSK2-DHNs might have arisen from an ancestral FSK3-DHNs.

We found a total of 101 DHNs containing one to three copies of the Y-segment per sequence at a N-terminal position, all of them in angiosperms. The mayority of the proteins belong to the YnSKn subgroup, while sequences lacking the S-segment (YnKn) only represent 15%. In both monocots and dicots we found YSKn and Y2SKn-DHNs, while Y3SKn- and YnKn-DHNs seem to be restricted to dicots alone (Fig. 2b; Supplementary Figs. S4–S6). Strikingly, the Phi-segment detected in our database corresponds to the N-terminal region of the previously defined GT-segment^7^ and is highly conserved in YSKn monocots.

We identified a total of 62 KS-DHNs in plant genomes, all of which share a novel N-terminal motif (H-segment, see below). Interestingly, the KS-HMM profile allowed us to identify KS-DHNs in the genome of the lycophyte *S. mollendorfii* and the gymnosperm *G. biloba*, but no KS-DHNs were identified in the genomes of the conifers *P. abies* and *P. glauca*. The proteins present a typical arrangement of KS motifs, with a K-segment followed by a lysine-rich stretch (B-segment) and a S-segment characteristic of this structural subgroup (S2-segment) (Fig. 3). This is the first time that KS-DHNs are identified in non-angiosperm species and indicates that this group of DHNs arose early in land plant evolution.

In angiosperms, all species analysed possessed one or two KS-DHNs genes with the exception of *Glycine max*, with four genes, and two Malpighiales species, *Salix purpurea* and *Populus trichocarpa*, with six and three KS-DHNs, respectively. The Malpigiales proteins are unique since they contain multiple K-segment repeats interspersed with glycine-rich sequences (Phi-segment) and the S2-segment is absent (Supplementary Fig. S8).

Concerning the Kn- and SKn-DHN structural subgroups, their representation in vascular plants was minor and, ultimately, they are phylogenetically related to other DHN structural subgroups (see below). In contrast, most of the eighteen non-vascular DHNs that we identified in the genomes of the mosses *P. patens* (six proteins), *Ceratodon purpureus* (six)*, Sphagnum fallax* (three) and the liverwort *Marchantia polymorpha* (three) belong to the Kn-structural subgroup. The exception is an atypical DHN containing a series of repetitive motifs resembling the Y-segment from *P. patens* (PpDHNA)^34^ and *C. purpureus* (Supplementary Fig. S10). A phylogenetic analysis indicates the presence of five DHN orthologue groups in *P. patens* and *C. purpureus* (Supplementary Figs. S9–S10), which reflects the phylogenetic proximity of the Funariidae and Dicranidae clades^35^, while the DHNs from *S. fallax* (Sphagnophytina) did not cluster with the other mosses. The DHNs of the liverwort *M. polymorpha* do not display any obvious homology to moss DHNs outside the K-segment.

### The H-segment is a novel conserved motif present in all KS-dehydrins

Our MEME analysis identified a highly conserved motif, not previously described, at the N-terminal region of all angiosperm KS-DHNs analysed. This 15-residue segment is characterized by a combination of hydrophobic amino acids Ile and Leu with amphipathic amino acids Lys and Glu, framed by two Gly at positions 3 and 15 conserved in 91% and 87% KS-DHNs, respectively (Fig. 2). In addition, there is high conservation of Lys (97%) and Ile (97%) in the central positions 7 and 8. Ile residues at positions 4 and 5 are less conserved, and are often replaced by other hydrophobic amino acids like Phe, Val or Met. Two His residues are found in positions 6 and 13 in 56% and 77% of KS-DHNs, respectively. KS-DHNs are characterized by sequences enriched in His amino acids, as reflected in the name HIRD11 for the *A. thaliana* KS-DHN, which stands for Histidine-Rich Domain 11 kDa^36^. Since this novel motif seems to be a signature of KS-DHNs, we propose to name it the H-segment**,** reflecting the particular nature of these kind of proteins.

**Figure 4:**
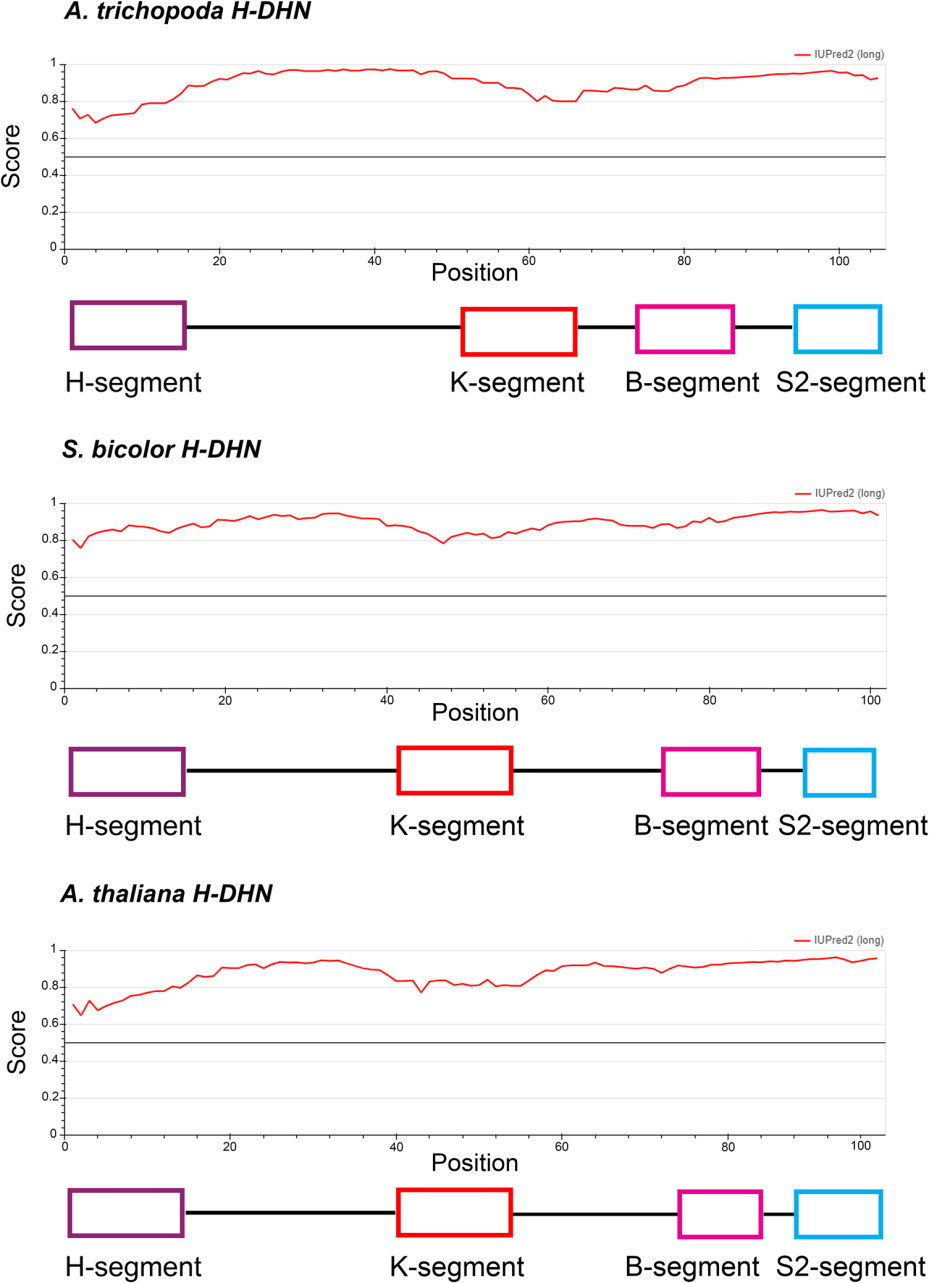
Disorder prediction plots for representative angiosperm H-DHNs (KS-DHN). Disorder of *A. trichopoda*, *S. bicolor* and *A. thaliana* H-DHN proteins were predicted by the IUPred2A program. Note that all proteins are predicted as fully disordered (score > 0.5), however, regions corresponding to the H- and K-motifs display reduced disorder tendency. The H-, K-, B and S-segments are indicated.

Intrinsically disordered proteins (IDPs), like dehydrins, have no single well-defined tertiary structure under native conditions. Using IUPred2A software we identified disordered protein regions in representative angiosperm KS-DHNs (H-DHNs) (Fig. 4). Interestingly, the regions spanning across the H- and K-segments correspond to the unique two regions that display reduced disorder tendency.

The K- and S-segments of KS-DHNs present some particular characteristics compared to FSK and YSK-DHNs (Fig. 2a and Supplementary Figs. S2–S7). Position 15 is occupied by Ile in almost all KS-DHNs instead of the Lys that it is typically present in the other DHNs. Even though Ile and Leu amino acids are generally considered conservative, there is evidence that these amino acids are not always interchangeable, affecting the affinity and specificity of protein–protein and protein-membrane interactions^37^, which might potentially lead to functional diversification of the KS-DHNs by modulating K-segment behaviour. In contrast to other DHNs, KS-dehydrins do not show a clear prevalence of Pro at position 16; instead, His is the most frequent amino acid at this position. In spite of these differences, the predicted α-helix structure delimited by conserved Gly amino acids of the K-segment is conserved in KS-DHNs (Fig. 4).

As for S-segments, which are characterised by a stretch of Ser residues, there are differences in the length of the Ser-amino acid stretch and neighbouring amino acids between KS-DHNs and other DHNs. The core of 6 to 9 Ser residues usually ended with negatively-charged Asp or Glu amino acids in all structural subgroups of DHNs. On the other hand, the triad Leu-His-Arg that precedes the Ser stretch, which is highly conserved in all FSK-DHNs and in the mayority of YSK-DHNs, is not found in KS-DHNs. Figure 2a shows the S-segment consensus for FSK and YSK-DHNs (segment S1) and the one found in KS-DHNs (segment S2, see also the alignments in Supplementary Figs. S2 and S7). The S-segment of all types of DHNs has been shown to be a hotspot for phosphorylation by kinases^8,38,39^, and the differences between the S1- and S2-segment could result in different kinase specificities. For instance, the triad Leu-His-Arg constitutes part of the recognition sequence for SnRK2 kinases^40^, which have been recently demonstrated to phosphorylate *A. thaliana* dehydrins ERD4 and ERD10 in response to osmotic stress^41^.

**Figure 5:**
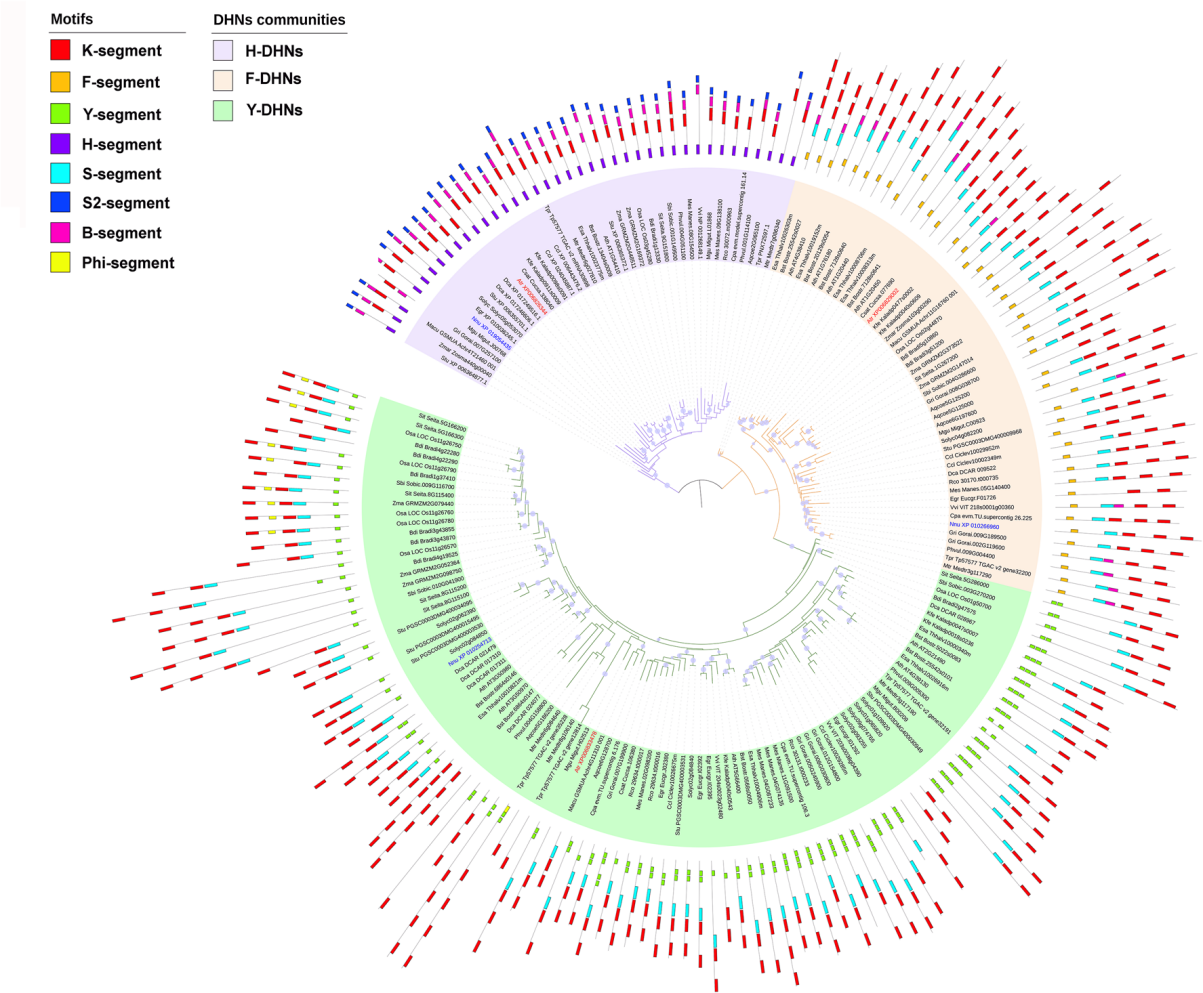
Unrooted phylogenetic tree of angiosperm DHN proteins. Amino acid sequences of DHN proteins from angiosperms were aligned and an approximately maximum-likelihood reconstruction of the phylogenetic relationships was generated using IQ-TREE. Branches with bootstrap support over 90% are indicated by a violet dot. The motifs of each protein are indicated by coloured boxes, as indicated. Note that all H-DHNs (KS-DHNs) are grouped together in a branch with high support, and most F- and Y-DHNs are also grouped together, forming three groups. DHN proteins from the angiosperm *A. trichopoda* (red) and the eudicot *N. nucifera* (blue) are indicated to show that they possess one DHN in each group. The tree is unrooted and shows the relative relatedness of the sequences, meaning that the position of the apparent root is arbitrary.

A 11-residue Lys-rich motif has been consistently detected in all KS-DHNs as well as in all FSK2 and the majority of FSK3-DHNs (Supplementary Figs. S1–S3; Fig. 5). In KS- and FSK-DHNs, the Lys-rich motif is located between the S-segment and the K-segment while, at the same position, YK- and YSK-DHNs usually have a RRKK or RRKKK sequence framed by Gly residues, a motif that resembles monopartite nuclear localization signals^42,43^. In conclusion, the KS-DHNs can be better described as having a H–K–S structural organization, with H being a newly described segment, exclusively present in this group of DHNs.

### Phylogenetic analysis reveals three basic groups of DHNs in angiosperms

Having identified the motifs that characterize the KS-structural subgroup, we sought to infer the phylogenetic relationships between the DHN proteins identified. We decided to use only angiosperm DHNs to build a phylogenetic tree due to the sparse taxonomic sampling of other land plant DHNs, as well as the inherent difficulty of aligning DHN sequences from more distant species, which have more divergent domain structures. We used the maximum-likelihood principle as implemented in IQ-TREE^23^ and estimated statistical robustness with the bootstrap method.

The resulting tree is roughly organised in three branches or groups (Fig. 5). All KS-DHNs, characterised by the presence of the H-segment (H-DHNs), are grouped together in a branch with high bootstrap support (100%). The other DHNs are separated into two branches, one harbouring most DHNs that contain the F-segment (FSKn), while the other contains DHNs carrying the Y-segment (YnSKn, YnKn). Interestingly, the few DHNs that contain only the K-segment or a combination of K- and S-segments are placed within the F or Y branches, indicating that these DHNs actually belong to either of these groups. Overall, the phylogenetic tree suggests that each angiosperm DHN belongs to one of three phylogenetic families, basically distinguished by the presence of the H-, F- or Y-segments. This conclusion is reinforced by the observation of the DHNs of plants at key phylogenetic positions. Thus, *Amborella trichopoda*, which belongs to a sister group to all other angiosperms (a “basal” angiosperm), possesses three DHNs, each one belonging to the H, F or Y groups (Fig. 5). Similarly, the three DHNs from *Nelumbo nucifera*, which belongs to a sister group to all eudicots, are also each one placed into the H, F and Y groups. Overall, the phylogenetic results suggest that these three groups of DHNs were present since the begining of angiosperm evolution.

### H-DHNs belong to a separate synteny community in angiosperms

Even though the phylogenetic tree described above separates angiosperm DHNs into three groups, the large number of different motifs and their divergent arrangement in DHNs makes the sequences difficult to align and reduces the certainty of the phylogenetic reconstruction. Since synteny analyses of orthologue genes can give important hints about the evolution of genomes and gene families^44^, we performed an analysis of the genomic neighbourhood (microsynteny) of DHN genes in order to reinforce our phylogenetic results.

**Figure 6:**
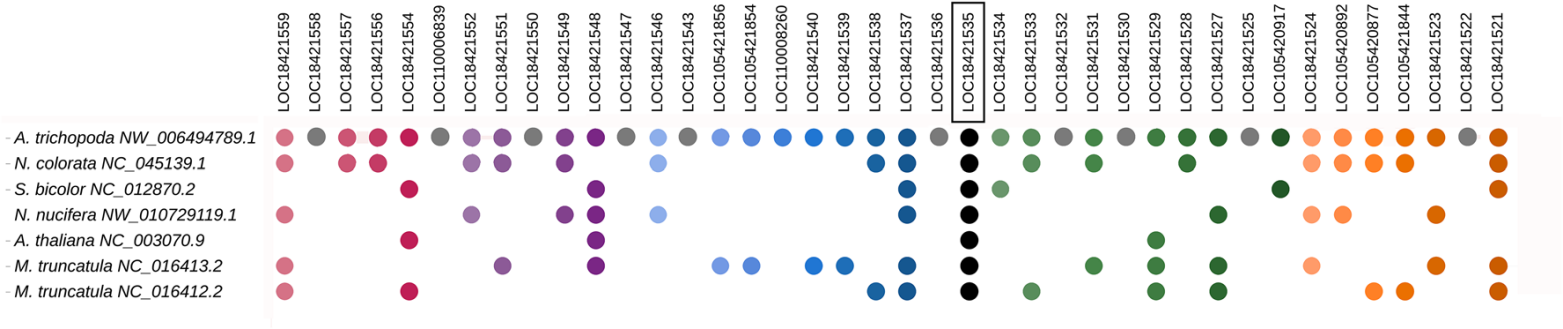
Microsynteny analysis of angiosperm H-DHNs genes. The genomic neighbourhood of the H-DHN gene of *A. trichopoda* (LOC18421535) is compared to that of other angiosperms. H-DHN genes are indicated as black dots, and a colour code indicates homologous genes present in the other species. Grey dots indicate genes only present in *A. trichopoda*. Some intervening genes in species other than *A. trichopoda* are not shown for clarity.

Recently, Artur et al.^17^ analysed DHN genes plant genomes and identified two main synteny blocks (or communities) among angiosperms, corresponding to DHNs containing the F (community 1) and Y (community 2) motifs. Their analysis, however, did not include DHNs of the KS group, presumably due to the difficulty of retrieving these sequences using the Pfam motif PF00257. To verify whether DHNs containing the H-segment would also be part of a syntenic community, we compared 40 genes surrounding the unique H-DHN of the basal angiosperm, *A. trichopoda* to the genomic neighbourhoods of H-DHN loci of the waterlily *Nymphaea colorata*^45^, the basal eudicot sacred lotus, *N. nucifera*^46^, the legume *Medicago truncatula*^47^, the model plant *A. thaliana* (HIRD11)^48^ and the monocot grass *Sorghum bicolor*^49^. All of these species possess only one H-DHN paralogue except for *M. truncatula*, which has two (Supplementary Data S1). We chose *A. trichopoda* as the basis for synteny comparison since this flowering plant belongs to a sister lineage to all other angiosperms (Amborellales), did not undergo the whole genome duplications that affected other lineages and its genome exhibits conserved synteny with other angiosperms^50,51^. As shown in Fig. 6, 17 genes that surround the H-DHN of *A. trichopoda* (LOC18421535) are also present around the H-DHN gene of *N. colorata*, which belongs to a group, the Nymphaeales, that is a sister lineage to all angiosperms except for Amborellales^52^. A smaller number of conserved genes are present around the H-DHN genes from the eudicots *N. nucifera*, *A. thaliana* (*HIRD11*) and *M. truncatula* and the monocot *S. bicolor* (Fig. 6). The microsynteny of H-DHN genes of other angiosperms is likewise conserved (not shown). H-DHNs possess two exons, with the whole coding region contained within the first exon and the second exon being no-coding, while F- and Y-DHNs usually have two coding exons (not shown). The conserved exon–intron structure also points to a common origin of H-DHNs. In conclusion, the microsynteny of H-DHN genes is conserved in angiosperms, indicating their true orthologous status and common evolutionary origin.

Along with one H-DHN gene, the genome of *A. trichopoda* contains two other DHN genes (LOC18424350 and LOC18426770). As mentioned above, in our phylogenetic tree, LOC18424350 is grouped together with F-DHNs and LOC18424350 with Y-DHNs (Fig. 5). Curiously, the F and Y motifs of these proteins are quite degenerated and are not readily recognised by the MEME program. A comparison of the genomic neighbourhoods of LOC18424350 and LOC18426770 of *A. trichopoda* with F- and Y-DHNs of *N. colorata* and *N. nucifera*, which likewise have only three DHN genes, reveals microsynteny conservation around these genes (Supplementary Data S2), confirming that LOC18424350 and LOC18426770 belong to the F- and Y-DHN synthenic communities, respectively.

In summary, it is apparent that DHN genes of angiosperms can be generally divided into three syntenic communities, each one characterised, among other features, by the presence of the H, F or the Y motif. We propose that these orthologous groups be called F-dehydrins (community 1), Y-dehydrins (community 2) and H-dehydrins (community 3). The presence of only three dehydrin genes in the genomes of plants belonging to groups that diverged early during angiosperm evolution, like *Amborella* and *Nymphaea*, or early at eudicot evolution, like *N. nucifera*, suggests that the genomes of the first flowering plants had one H-, F- and Y-dehydrin gene each. Subsequent whole genome duplications in eudicots and monocots greatly increased the repertoire of these genes, especially those encoding F- and Y-dehydrins.

### Each DHN orthologous group presents distinctive hydrophylin biochemical properties

**Figure 7:**
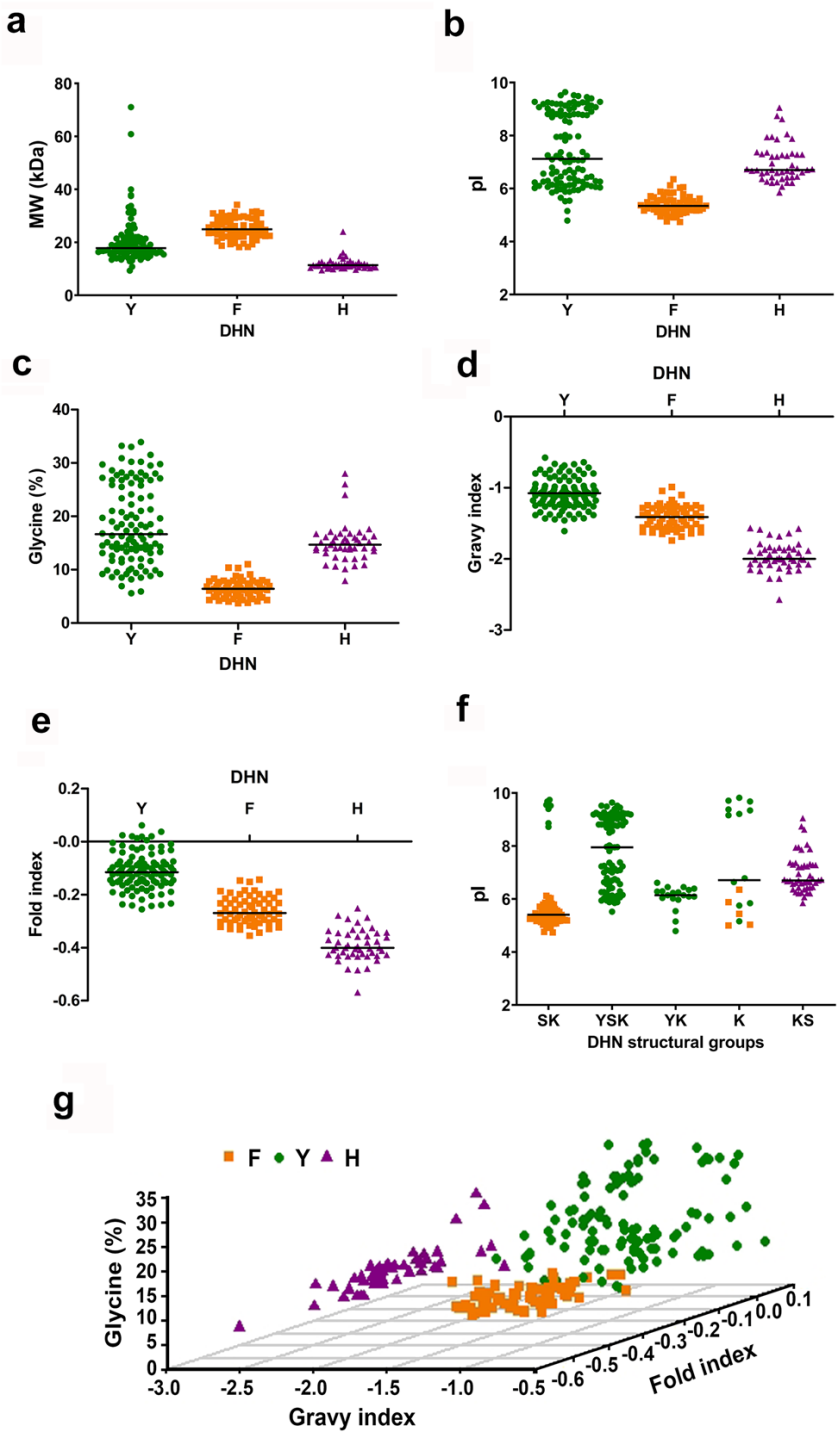
Distribution of biochemical and biophysical properties of angiosperm DHNs. Scatter plots show the distribution of molecular weight (**a**), isoelectric point (**b** and **f**), glycine content (**c**), GRAVY index (**d**), Fold Index (**e**) or glycine content, GRAVY and Fold index simultaneously (**g**) in orthologous or structural subgroups of DHNs. Members of the three orthologous groups of DHNs are colour-coded: Y- (green), F- (orange) and H-DHNs (violet).

We compared the biochemical and biophysical properties of angiosperm DHNs from the H-, F- and Y-orthologous groups, namely features such as molecular weight (MW), isoelectric point (pI), as well as parameters related to the hydrophilin character of the proteins (Supplementary Data S1). Each DHN orthologous group has a different MW distribution, with a characteristic statistical median (Fig. 7a). H-DHNs are the smallest DHNs with the narrowest range of MW (10–16 kDa), reflecting that the number of residues and domain structure in this group are relatively constant. F-DHNs also have a compact MW distribution (18–35 kDa), while Y-DHNs present a main subgroup of proteins with 10–25 kDa and a number of DHNs over 30 kDa that belong to monocots. The high MW of this latter subgroup is not due to an increased number of conserved Y or K domains, but to the presence of long Gly-rich regions (Supplementary Figs. S4–S6). Most H-DHNs present acidic pI values, with neutral and basic isoforms being found in some species (Fig. 7b). F-DHNs have a very homogeneous acidic pI profile, with a unimodal distribution between 5 and 6. In contrast, Y-DHNs display a bimodal distribution consisting of two main subgroups of DHNs with basic and acid pI values, and a smaller subgroup with pI values close to neutrality.

As for glycine content, both F- and H-DHNs present a compact and homogeneous distribution of percentage of glycine residues (Fig. 7c). The DHNs with the lowest percentage of glycine (around 10%) are the F-DHNs. Remarkably, DHNs of the FSK3 structural subgroup are characterized by the presence of many proline stretches, which might play an equivalent role to that of glycine in terms of the disruption of the protein structure (Supplementary Figs. S2–S3). Notably, a larger dispersion in the percentage of glycine residues is observed in Y-DHNs (5–35%).

All DHNs display a negative GRAVY index, showing the characteristic hydrophilicity of these proteins (Fig. 7d). The Y-DHNs include the least hydrophilic proteins, while H-DHNs are the most hydrophilic, with GRAVY indexes ranging from -2.8 to -1.3. The atypical H-DHNs from *S. purpurea* and *P. trichocarpa* (Malpighiales) are the least hydrophilic in this group, with an index around -1.3. The scores for F-DHNs have an intermediate range, overlaping with the other groups. We also evaluated the Fold Index of DHNs using the FoldIndex algorithm^53^. The fold index of DHNs shows a similar distribution to that of the GRAVY index, with H-DHNs being the most intrinsically unfolded, while F- and Y-DHNs have a less unfolded character (Fig. 7e).

When analysing the pI distribution in the five traditional DHN structural subgroups, it can be noted that the bimodal character observed in SK- and K-type DHNs strongly correlates with their evolutionary origin (Fig. 7b). Thus, five of the K-type DHNs that display acidic pI are F-DHNs, while all SK- and K-DHNs with high pI values belong to the Y-DHN orthologous group (Fig. 7b, f). Similar observations can be made concerning other DHN characteristics (Supplemental Fig. S11), suggesting that orthologous groups are better indicators of the physicochemical character of DHNs than the structural subgroups^7^. In summary, in general terms, the biochemical and biophysical characteristics of DHNs correlate well with the three orthologous groups (Fig. 7g). Since these features are likely related to the function of DHNs, this suggests that functional studies should take into consideration this phylogenetic framework described here.

## Discussion

In this work, we present a phylogenetic framework that sheds light on the relationships of DHNs, especially in angiosperms. The main points of our work are: i) searches of DHN in plant genomic databases need to be done with a combination of HHM profiles to retrieve all types of DHNs; ii) KS-DHNs possess a new, conserved structural domain present at the N-terminus, the H-domain; iii) phylogenetic and synteny analyses show that all angiosperm DHNs can be subdivided into three DHN orthologous groups, distinguished by the presence of the H-, F- or Y-domains, and iv) the psychochemical characteristics of DHNs correlate with the orthologous groups, indicating that the evolutionary origin of DHNs should be taken into consideration when studying their function.

The reconstruction of the evolution of DHNs is a complex task, due to the modular nature of these proteins, characterized by the presence of short conserved segments surrounded by less conserved sequences. Thus, the coupling of phylogenetic reconstruction with microsynteny analyses was crucial for determining the evolutionary relationships between DHNs. Angiosperm DHNs are divided into three orthologous groups, H-DHNs, F-DHNs and Y-DHNs, which can, in most cases, be readly recognised by the presence of the H-, F- or Y-segments. All angiosperms analysed by us possess at least one DHN member of each homologous group, including *A. trichopoda* and *N. colorata*, which belong to sister groups to other angiosperms, indicating that the first angiosperms had genes enconding the three types of DHNs. Synteny analyses could not be extended to non-angiosperm species due to the fast rate of synteny loss that is typical for plants^44,54^. H-DHNs have two exons, with the whole coding region within exon 1, and this conserved exon–intron structure also points to their common origin. It has been described that introns of dehydrin genes are frequently present within the region encoding the S-segment^55^. Interestingly, the S-segment of H-DHNs is located at the C- terminus of the protein, and the intron is next to the stop codon in exon 1.

Our analysis indicated that H-, F- and Y-DHNs are clearly distinguished from each other in features that characterize hydrophilins and intrinsically-disordered proteins (IDPs). All dehydrins of K and SK-structural subgroups actually belong to the F- or Y- syntenic groups, indicating that a classification based solely on segment composition ends up grouping DHNs with very different physicochemical properties^7^.

The diversity of DHNs is not encompassed by the HMM model usually employed to search for DHN genes, namely Pfam00257. Indeed, we show here that most H-DHNs are not recognized by this model, which might be the reason that genome-wide analyses of DHNs generally fail to retrieve many members of this group^13,15,17^. In the recent work by Artur et al.^17^, the underestimation of KS-DHNs led to the definition of only two groups of orthologous genes (Y and F-DHNs), missing the existence of a third group (H-DHNs). Genome-wide analyses of DHNs have also been carried out using the BLASTP program, but previous works using this search tool were not able to detect distant orthologous genes like *S. mollendorffii* and *G. biloba* H-DHNs^7,16^. In view of this, we propose that studies aimed at identifying DHNs should use HMM profiles based on H-, F- and Y-DHNs separately in order to pinpoint all members of this protein family.

Importantly, we describe that KS-DHNs possess a new motif that we named the H-segment, due to the presence of two conserved His residues. This segment is always located at the N-terminus of the proteins, thus KS-DHNs can be better described as bearing a H–K–S organization of motifs. As mentioned above, phylogenetic and synteny analyses indicate that angiosperm DHNs are all evolutionarily related. The presence of DHNs with a distinct H–K–S organization in *S. moellendorffii* and *G. biloba* shows that H-DHNs appeared in the early evolution of tracheophytes. Although some KS-DHNs have been described before, our work is the first, to our knowledge, to provide a thorough description of this group of DHNs.

Several pieces of evidence demonstrated that dehydrins are intrinsically disordered proteins in solution, but despite this, they are able to gain structure when bound to some ligands^56^. Our analysis indicates that both H- and K-segments present in angiosperm H-DHNs correspond to reduced disordered regions. On the other hand, a structural prediction of *A. thaliana* and *Z. mays* KS-DHNs, obtained from AlphaFold protein structure database (https://alphafold.ebi.ac.uk/)^57^, indicates with low confidence the presence of a α-helix spanning both motifs. That should not be necessarily interpreted as indicating that the H-domain has a helical structure in native condition, instead, it might suggest that this region is able to form transient α-helix in the presence of a ligand as was demonstrated for K-segments^9,58^.

The best studied member of the H-DHN group is the HIRD11 protein from *A. thaliana*. AtHIRD11 is expressed ubiquitously, with somewhat higher levels in flowers^48^. HIRD11 binds to metal ions and can protect proteins from heavy metal damage^48,59^ and reduce free radical generation^60^. Yokoyama et al.^61^ have recently showed that both the K- and the H-segments (called K and NK1, respectively) of AtHIRD11 can protect proteins from freezing damage with similar efficiencies. Thus, K- and H-segments might play overlapping roles in the activity of H-DHNs. It has been reported that DHNs can bind in vitro to metal ions through histidine-rich motives^36^ and also a role in nuclear localizaton for an atypical track of histidines in the SK3-DHN of *Opuntia streptacantha* has been demonstrated^62^. Nevertheless, these histidine-rich motifs do not correspond to the H-domain, it will be interesting to study the functional implicance of histidine residues of the H-segment in the future.

In conclusion, we consider that the classification of angiosperm DHNs into three homologous groups, as proposed here, better reflects the diversity of DHNs and should complement the traditional classification into six structural subgroups in the study of the function of these proteins.

## Supplementary Information

Supplementary Information.

Supplementary Figures.
