# Supplementary material for: Evolutionary analysis of angiosperm dehydrin gene family reveals three orthologues groups associated to specific protein domains
Source: Sci Rep. 2021 Dec 13;11:23869. doi: 10.1038/s41598-021-03066-5 (PMC8669000; doi:10.1038/s41598-021-03066-5)
Supplement: Supplementary file 2 — Supplementary Figures. [file 41598_2021_3066_MOESM2_ESM.pdf]

Evolutionary analysis of angiosperm dehydrin gene family reveals three orthologues groups associated to specific protein domains

Alejandra E. Melgar[1,2] and Alicia M. Zelada[1,2*]

[1]Laboratorio de Agrobiotecnología, Departamento de Fisiología, Biología Molecular y Celular, Facultad de Ciencias Exactas y Naturales, Universidad de Buenos Aires, Buenos Aires, Argentina

[2]Instituto de Biodiversidad y Biología Experimental y Aplicada, Consejo Nacional de Investigaciones Científicas y Técnicas-Universidad de Buenos Aires (IBBEA, CONICET-UBA), Buenos Aires, Argentina

*Author for correspondence: azelada@fbmc.fcen.uba.ar

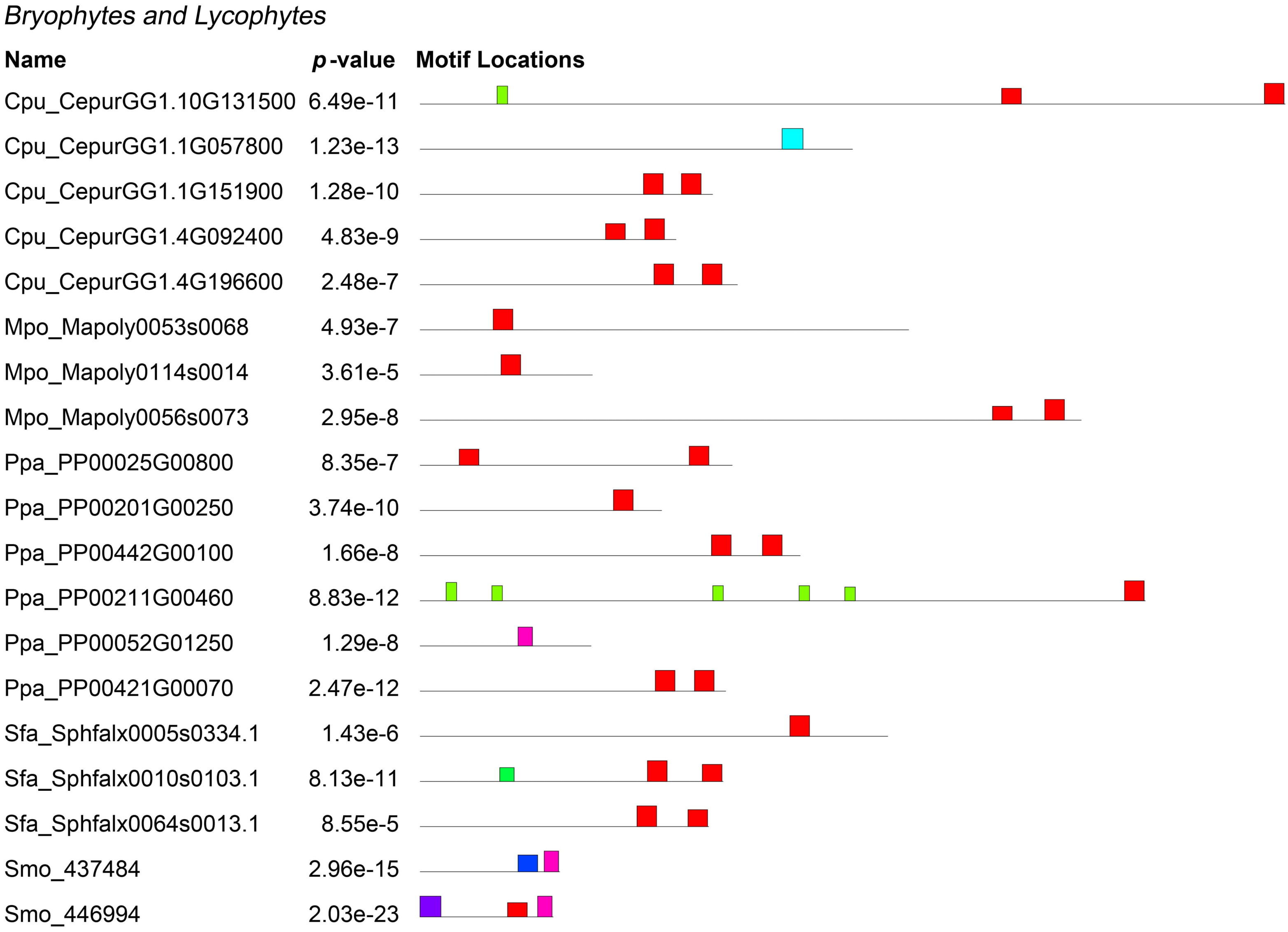
Bryophytes and Lycophytes
Name
p-value
Motif Locations
Cpu_CepurGG1.10G131500 6.49e-11
Cpu_CepurGG1.1G057800 1.23e-13
Cpu_CepurGG1.1G151900 1.28e-10
Cpu_CepurGG1.4G092400 4.83e-9
Cpu_CepurGG1.4G196600 2.48e-7
Mpo_Mapoly0053s0068 4.93e-7
Mpo_Mapoly0114s0014 3.61e-5
Mpo_Mapoly0056s0073 2.95e-8
Ppa_PP00025G00800 8.35e-7
Ppa_PP00201G00250 3.74e-10
Ppa_PP00442G00100 1.66e-8
Ppa_PP00211G00460 8.83e-12
Ppa_PP00052G01250 1.29e-8
Ppa_PP00421G00070 2.47e-12
Sfa_Sphfalx0005s0334.1 1.43e-6
Sfa_Sphfalx0010s0103.1 8.13e-11
Sfa_Sphfalx0064s0013.1 8.55e-5
Smo_437484 2.96e-15
Smo_446994 2.03e-23

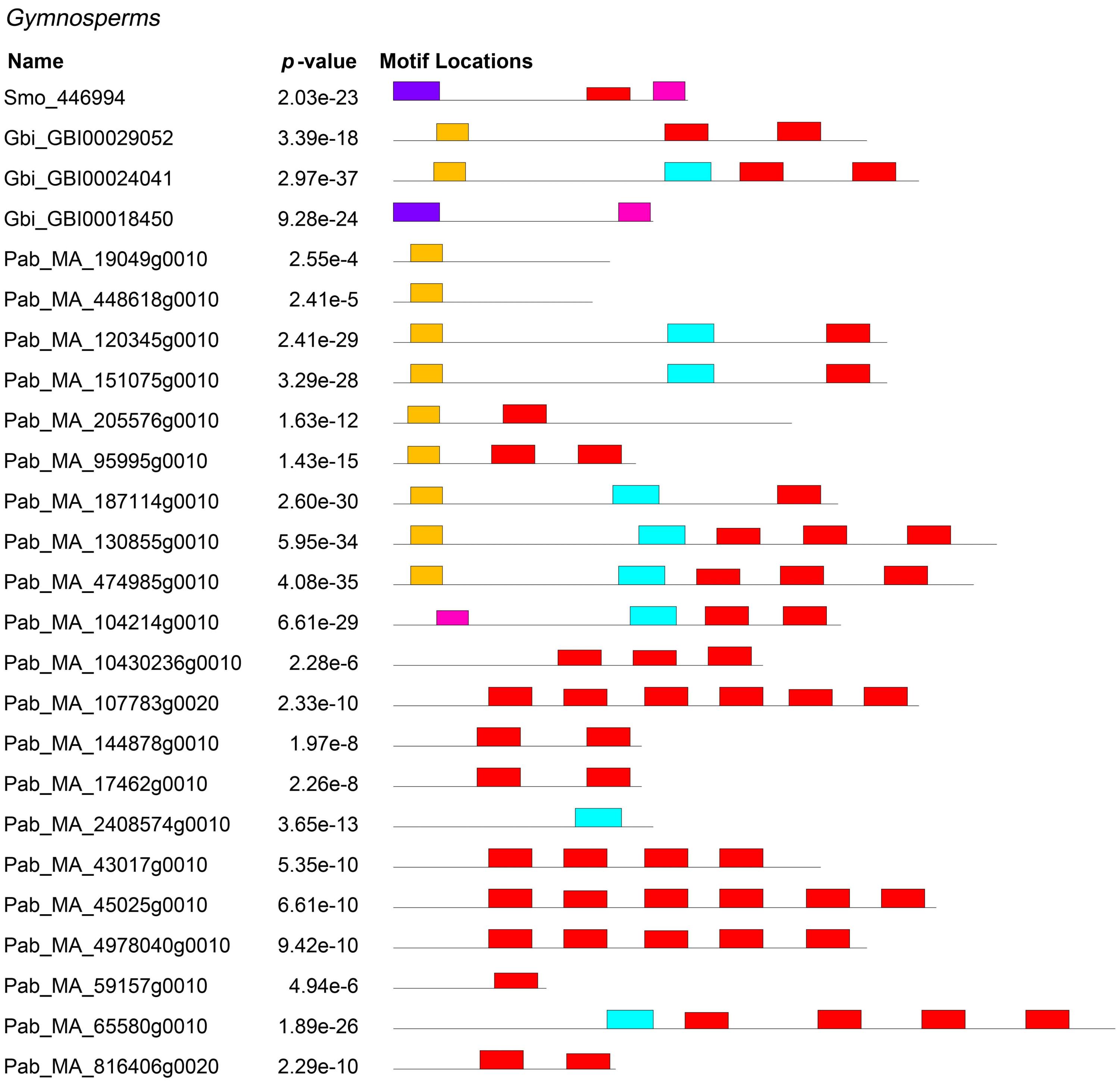
Gymnosperms
Name
p-value
Motif Locations
Smo_446994 2.03e-23
Gbi_GBI00029052 3.39e-18
Gbi_GBI00024041 2.97e-37
Gbi_GBI00018450 9.28e-24
Pab_MA_19049g0010 2.55e-4
Pab_MA_448618g0010 2.41e-5
Pab_MA_120345g0010 2.41e-29
Pab_MA_151075g0010 3.29e-28
Pab_MA_205576g0010 1.63e-12
Pab_MA_95995g0010 1.43e-15
Pab_MA_187114g0010 2.60e-30
Pab_MA_130855g0010 5.95e-34
Pab_MA_474985g0010 4.08e-35
Pab_MA_104214g0010 6.61e-29
Pab_MA_10430236g0010 2.28e-6
Pab_MA_107783g0020 2.33e-10
Pab_MA_144878g0010 1.97e-8
Pab_MA_17462g0010 2.26e-8
Pab_MA_2408574g0010 3.65e-13
Pab_MA_43017g0010 5.35e-10
Pab_MA_45025g0010 6.61e-10
Pab_MA_4978040g0010 9.42e-10
Pab_MA_59157g0010 4.94e-6
Pab_MA_65580g0010 1.89e-26
Pab_MA_816406g0020 2.29e-10

Angiosperms-Basal

Name	*p*-value
Atr_evm_27.TU.AmTr_v1.0_scaffold00001.295	6.29e-33
Atr_evm_27.model.AmTr_v1.0_scaffold00004.97	3.12e-36
Atr_evm_27.TU.AmTr_v1.0_scaffold00082.27	3.76e-30
Nco_Nycol.I01776	4.06e-35
Nco_Nycol.J01522	2.65e-26
Nco_Nycol.B01145	4.32e-25
Nco_Nycol.B00068	2.28e-33

Motif Locations

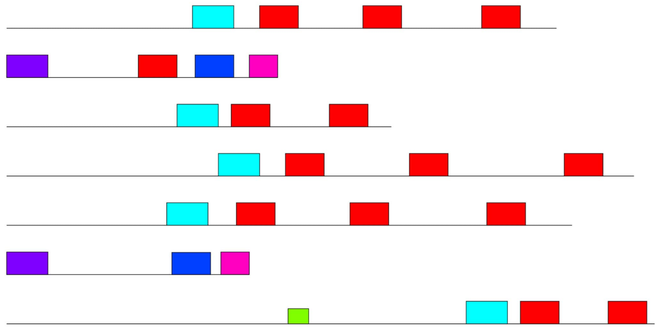

Angiosperms-Monocots

Name	*p*-value
Macu_GSMUA_Achr11G16760_001	1.51e-37
Macu_GSMUA_Achr4T21460_001	6.05e-34
Macu_GSMUA_Achr4G11310_001	3.29e-27
Aco_Aco011968	9.77e-40
Aco_OAY73247.1	3.69e-33
Aco_Aco016518	3.76e-35
Aco_Aco016515	3.46e-35
Aco_Aco021122	2.53e-15
Aco_Aco021124	2.59e-25
Aco_Aco016516	7.18e-29
Zmar_Zosma103g00290	1.05e-29
Zmar_Zosma440g00040	4.62e-40
Bdi_Bradi3g51200	5.44e-40
Bdi_Bradi5g10860	8.92e-40
Bdi_Bradi1g13330	1.97e-34
Bdi_Bradi2g47575	2.72e-34
Bdi_Bradi4g19525	4.35e-35
Bdi_Bradi1g37410	4.24e-41
Bdi_Bradi3g43855	2.44e-39
Bdi_Bradi3g43870	8.80e-39
Bdi_Bradi4g22280	9.20e-27
Bdi_Bradi4g22290	8.16e-29
Osa_LOC_Os02g44870	5.48e-40
Osa_LOC_Os03g45280	2.66e-36
Osa_LOC_Os01g50700	6.14e-31
Osa_LOC_Os11g26570	1.26e-35
Osa_LOC_Os11g26750	6.04e-38
Osa_LOC_Os11g26760	2.80e-43
Osa_LOC_Os11g26780	3.58e-43
Osa_LOC_Os11g26790	9.69e-42

Motif Locations

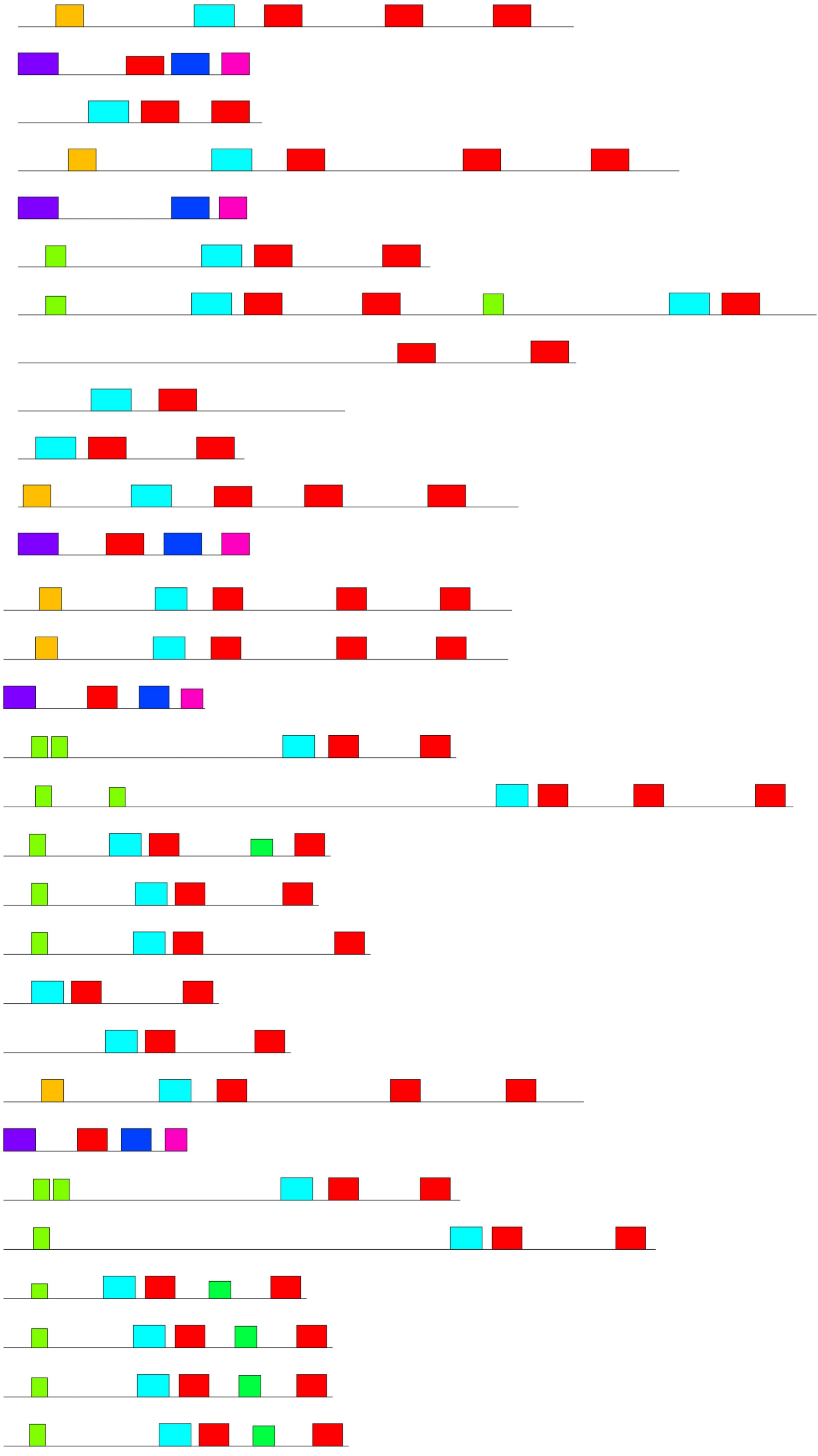

(Continue to next page)

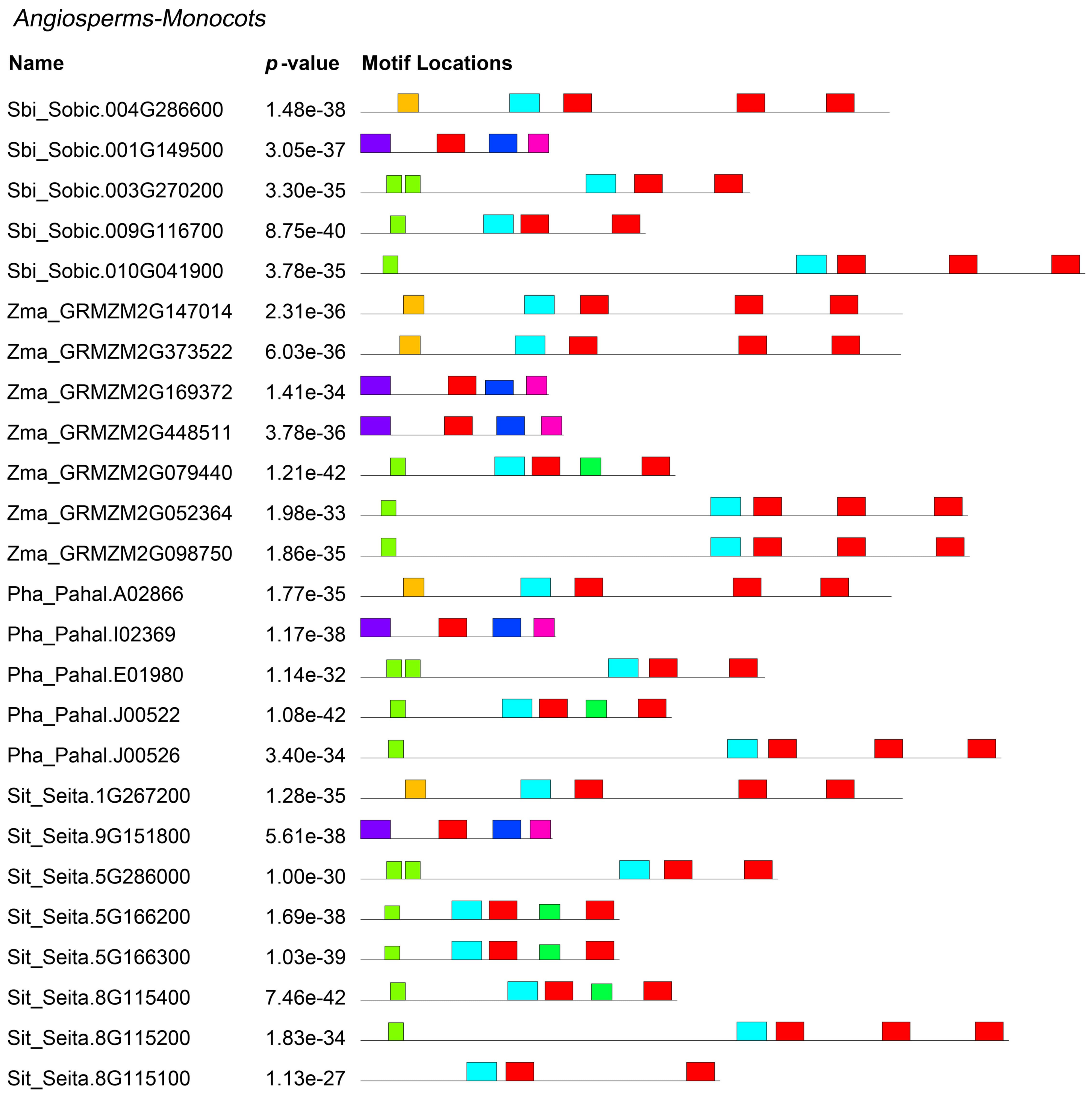
Angiosperms-Monocots
Name
p-value
Motif Locations
Sbi_Sobic.004G286600 1.48e-38
Sbi_Sobic.001G149500 3.05e-37
Sbi_Sobic.003G270200 3.30e-35
Sbi_Sobic.009G116700 8.75e-40
Sbi_Sobic.010G041900 3.78e-35
Zma_GRMZM2G147014 2.31e-36
Zma_GRMZM2G373522 6.03e-36
Zma_GRMZM2G169372 1.41e-34
Zma_GRMZM2G448511 3.78e-36
Zma_GRMZM2G079440 1.21e-42
Zma_GRMZM2G052364 1.98e-33
Zma_GRMZM2G098750 1.86e-35
Pha_Pahal.A02866 1.77e-35
Pha_Pahal.I02369 1.17e-38
Pha_Pahal.E01980 1.14e-32
Pha_Pahal.J00522 1.08e-42
Pha_Pahal.J00526 3.40e-34
Sit_Seita.1G267200 1.28e-35
Sit_Seita.9G151800 5.61e-38
Sit_Seita.5G286000 1.00e-30
Sit_Seita.5G166200 1.69e-38
Sit_Seita.5G166300 1.03e-39
Sit_Seita.8G115400 7.46e-42
Sit_Seita.8G115200 1.83e-34
Sit_Seita.8G115100 1.13e-27

Angiosperms-Eudicots

**Name**	***p*-value**
Sit_Seita.8G115100	1.13e-27
Nnu_XP_010266960	2.24e-41
Nnu_XP_019054435	2.19e-27
Nnu_XP_010254713	2.01e-37
Aqcoe6G197600	1.59e-39
Aqcoe5G125000	1.55e-34
Aqcoe5G125200	2.61e-13
Aqcoe2G065100	2.80e-34
Aqcoe6G128700	1.07e-32
Aqcoe5G186200	9.20e-14
Dca_DCAR_009522	1.59e-45
Dca_XP_017246606.1	2.53e-26
Dca_XP_017249516.1	1.05e-26
Dca_DCAR_017311	3.61e-33
Dca_DCAR_017310	1.94e-33
Dca_DCAR_028967	4.77e-30
Dca_DCAR_024077	9.75e-11
Dca_DCAR_021479	7.18e-27
Mgu_Migut.C00923	3.50e-38
Mgu_Migut.J00768	2.34e-31
Mgu_Migut.L01868	1.82e-28
Mgu_Migut.H02513	5.83e-34
Mgu_Migut.B00209	1.77e-32
Stu_PGSC0003DMG400009968	8.93e-39
Stu_XP_006364877.1	5.13e-30
Stu_XP_006355701.1	3.29e-38
Stu_XP_006365372.1	5.39e-23
Stu_PGSC0003DMG400003531	3.92e-38
Stu_PGSC0003DMG400030949	1.54e-30
Stu_PGSC0003DMG400003530	1.42e-30
Stu_PGSC0003DMG400034095	5.64e-34
Solyc04g082200	5.53e-42
Solyc_Solyc05g053070	1.54e-37
Solyc02g084840	3.92e-38
Solyc01g109920	6.23e-32
Solyc02g062390	2.57e-29
Solyc02g084850	3.65e-30
Solyc01g065820	8.42e-11
Solyc02g093255	1.53e-11
Solyc09g074765	1.42e-10

Motif Locations

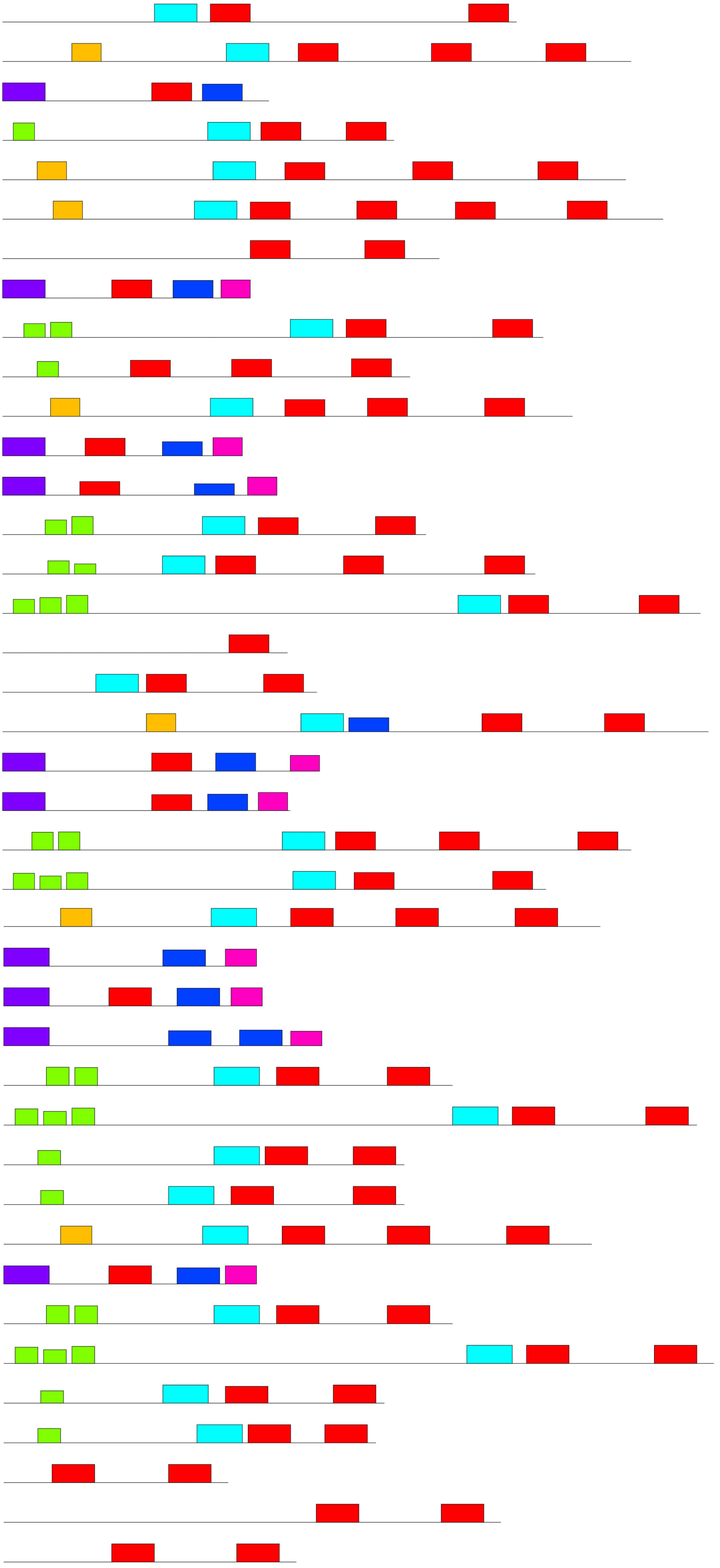

(Continue to next page)

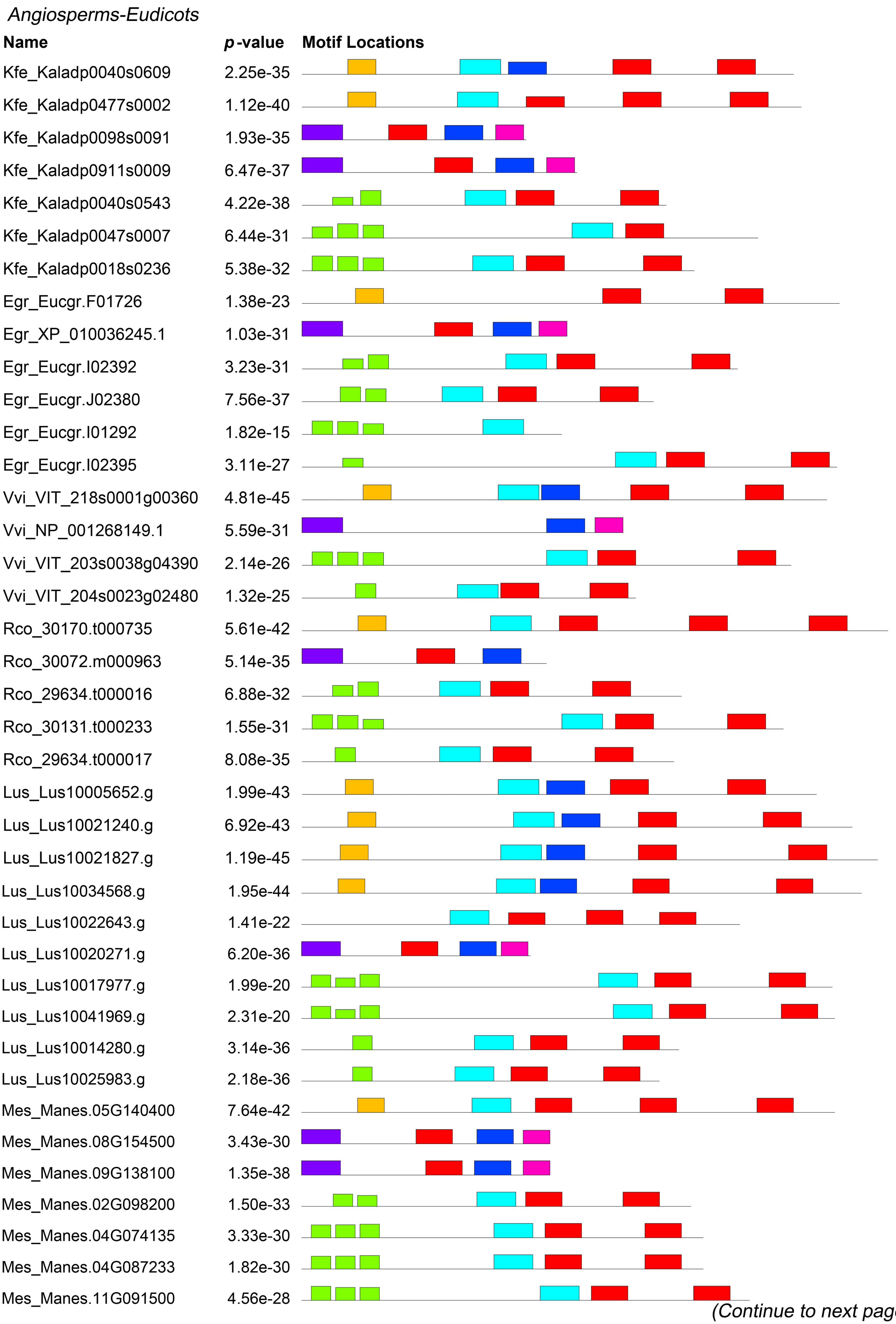

(Continue to next page)

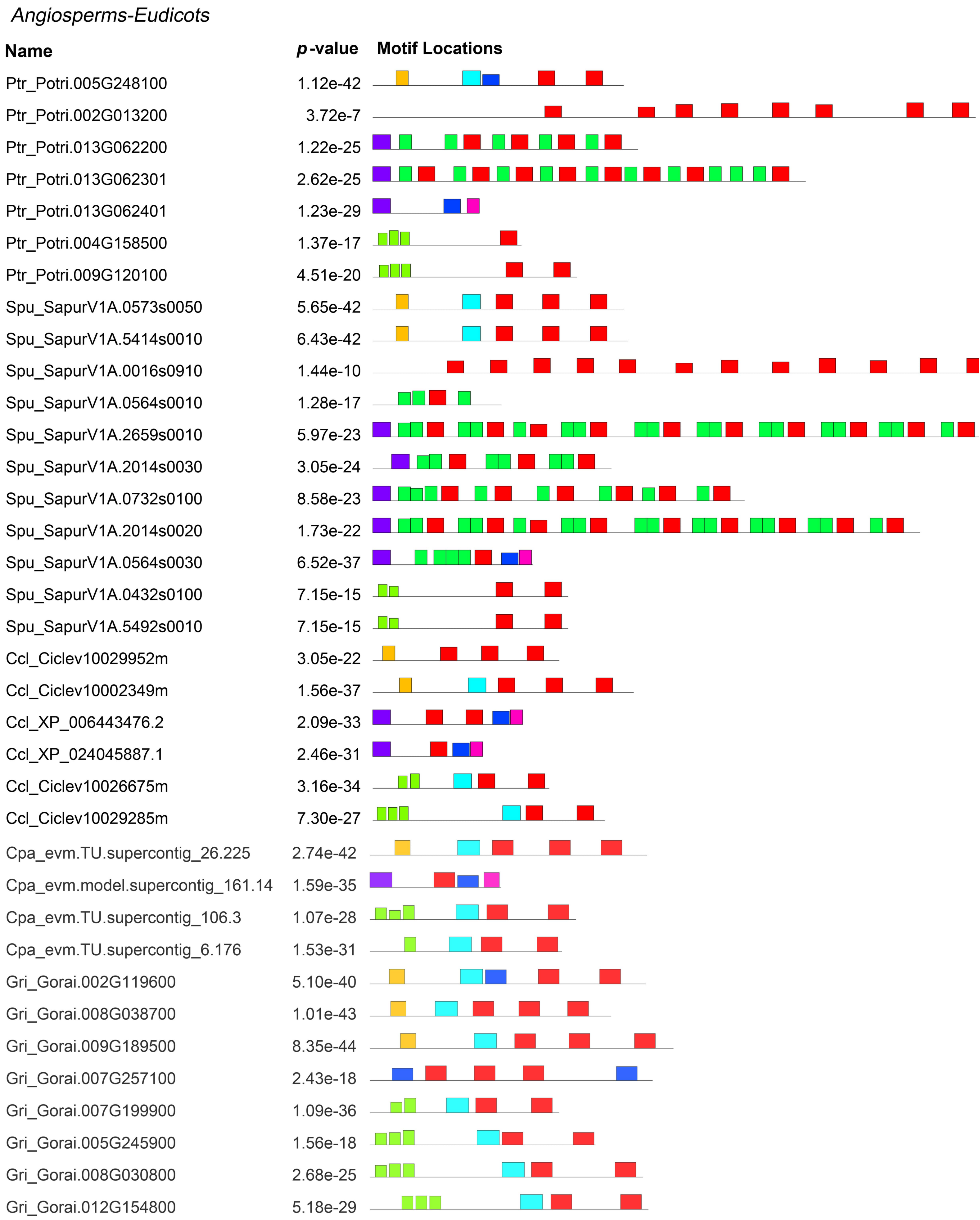

(Continue to next page)

Angiosperms-Eudicots

Name	*p*-value	Motif Locations
Ath_AT4G38410	4.06e-32	
Ath_AT1G76180	7.24e-45	
Ath_AT1G20440	1.84e-40	
Ath_AT1G20450	2.82e-45	
Ath_AT1G54410	3.70e-38	
Ath_AT4G39130	5.75e-15	
Ath_AT5G66400	4.23e-35	
Ath_AT2G21490	2.03e-33	
Ath_AT3G50980	1.60e-35	
Ath_AT3G50970	7.18e-8	
Aly_AL2G36240	3.06e-45	
Aly_AL7G10280	5.14e-29	
Aly_AL1G33370	1.16e-36	
Aly_AL1G33380	7.54e-47	
Aly_AL1G64750	3.75e-37	
Aly_AL7G11070	8.37e-15	
Aly_AL4G10674	2.71e-33	
Aly_AL8G45130	9.82e-35	
Aly_AL5G31400	6.12e-34	
Aly_AL5G31370	4.76e-8	
Aly_AL5G31390	3.62e-8	
Bst_Bostr.20129s0054	6.71e-46	
Bst_Bostr.25542s0027	7.89e-27	
Bst_Bostr.7128s0640	1.20e-38	
Bst_Bostr.7128s0641	4.31e-44	
Bst_Bostr.13404s0009	3.88e-37	
Bst_Bostr.0568s0050	7.35e-33	
Bst_Bostr.25542s0101	3.81e-13	
Bst_Bostr.5022s0083	3.12e-33	
Bst_Bostr.6864s0146	1.43e-28	
Bst_Bostr.6864s0147	4.06e-7	
Cgr_Cagra.0799s0088	5.18e-46	
Cgr_Cagra.1383s0028	3.39e-26	
Cgr_Cagra.25489s0001	3.22e-39	
Cgr_Cagra.25489s0002	9.15e-46	
Cgr_Cagra.27207s0001	1.96e-38	
Cgr_Cagra.2374s0005	1.86e-13	

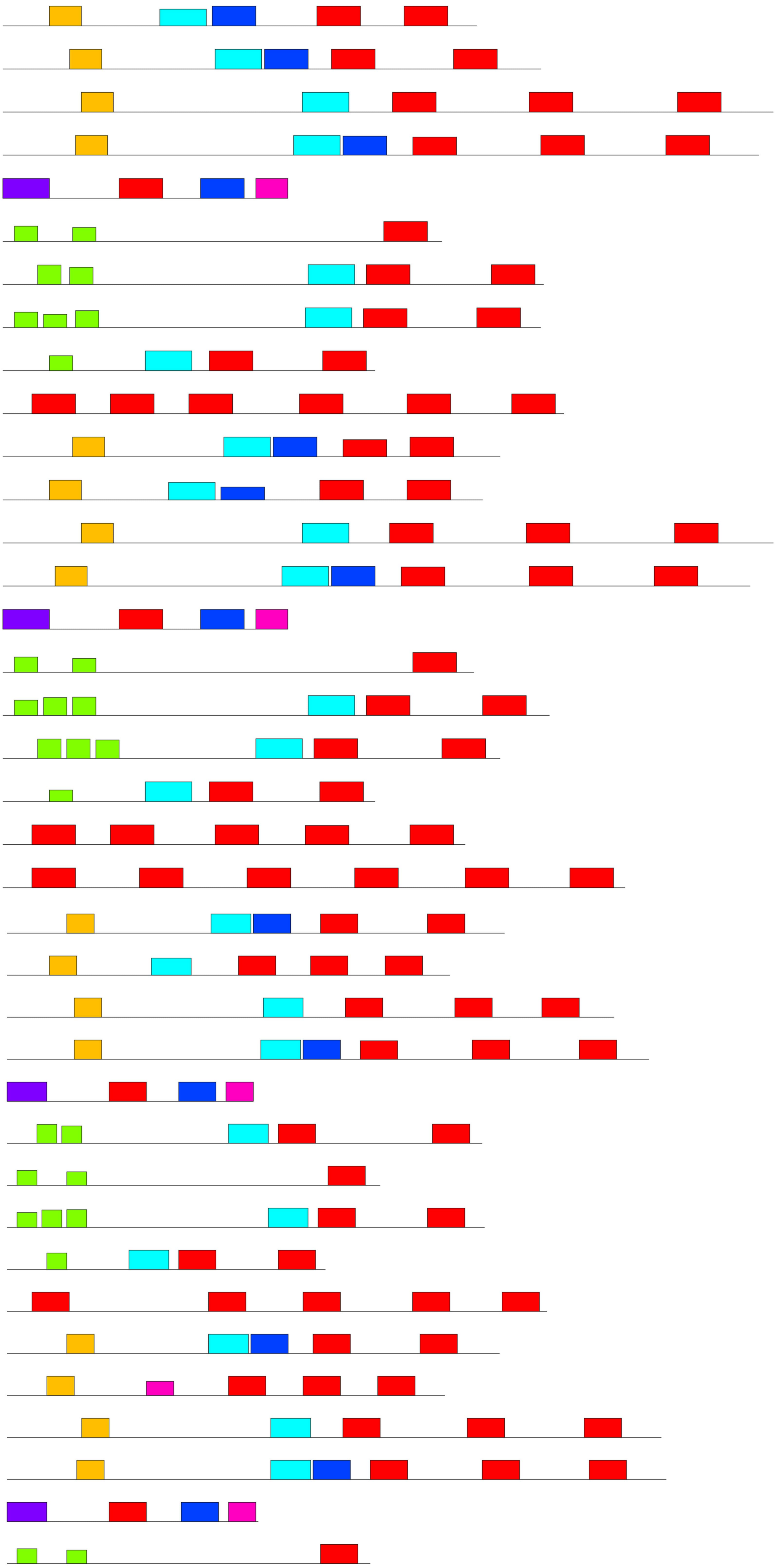

(Continue to next page)

Angiosperms-Eudicots

Name	*p*-value
Cgr_Cagra.2374s0005	1.86e-13
Cgr_Cagra.0926s0063	1.24e-34
Cgr_Cagra.0926s0062	1.52e-8
Cgr_Cagra.2007s0059	4.19e-29
Esa_Thhalv10019152m	5.44e-46
Esa_Thhalv10008313m	2.35e-44
Esa_Thhalv10008706m	1.41e-34
Esa_Thhalv10026303m	5.29e-32
Esa_Thhalv10023775m	9.28e-38
Esa_Thhalv10026916m	7.35e-17
Esa_Thhalv10004906m	3.31e-33
Esa_Thhalv10000340m	2.14e-33
Esa_Thhalv10010821m	1.95e-34
Csat_Cucsa.077690	3.03e-41
Csat_Cucsa.338040	7.28e-33
Csat_Cucsa.106380	2.51e-36
Gma_Glyma.04G009400	9.36e-45
Gma_Glyma.16G037900	7.85e-38
Gma_Glyma.16G038000	6.63e-33
Gma_Glyma.17G187600	2.27e-35
Gma_Glyma.19G114700	1.14e-34
Gma_Glyma.07G090400	5.70e-18
Gma_Glyma.09G185500	9.96e-20
Gma_Glyma.12G235800	7.67e-18
Gma_Glyma.13G201300	1.67e-24
Gma_Glyma.04G009900	3.60e-35
Gma_Glyma.08G048900	6.78e-11
Mtr_Medtr3g117290	9.05e-44
Mtr_Medtr6g027810	3.78e-38
Mtr_Medtr7g086340	6.76e-34
Mtr_Medtr6g084640	1.40e-20
Mtr_Medtr3g117190	7.56e-35
Mtr_Medtr8g106140	8.56e-12
Phvul.009G004400	4.94e-45
Phvul.001G114100	1.85e-35
Phvul.004G051100	1.53e-30
Phvul.004G158800	2.74e-16
Phvul.009G005300	3.84e-34

Motif Locations

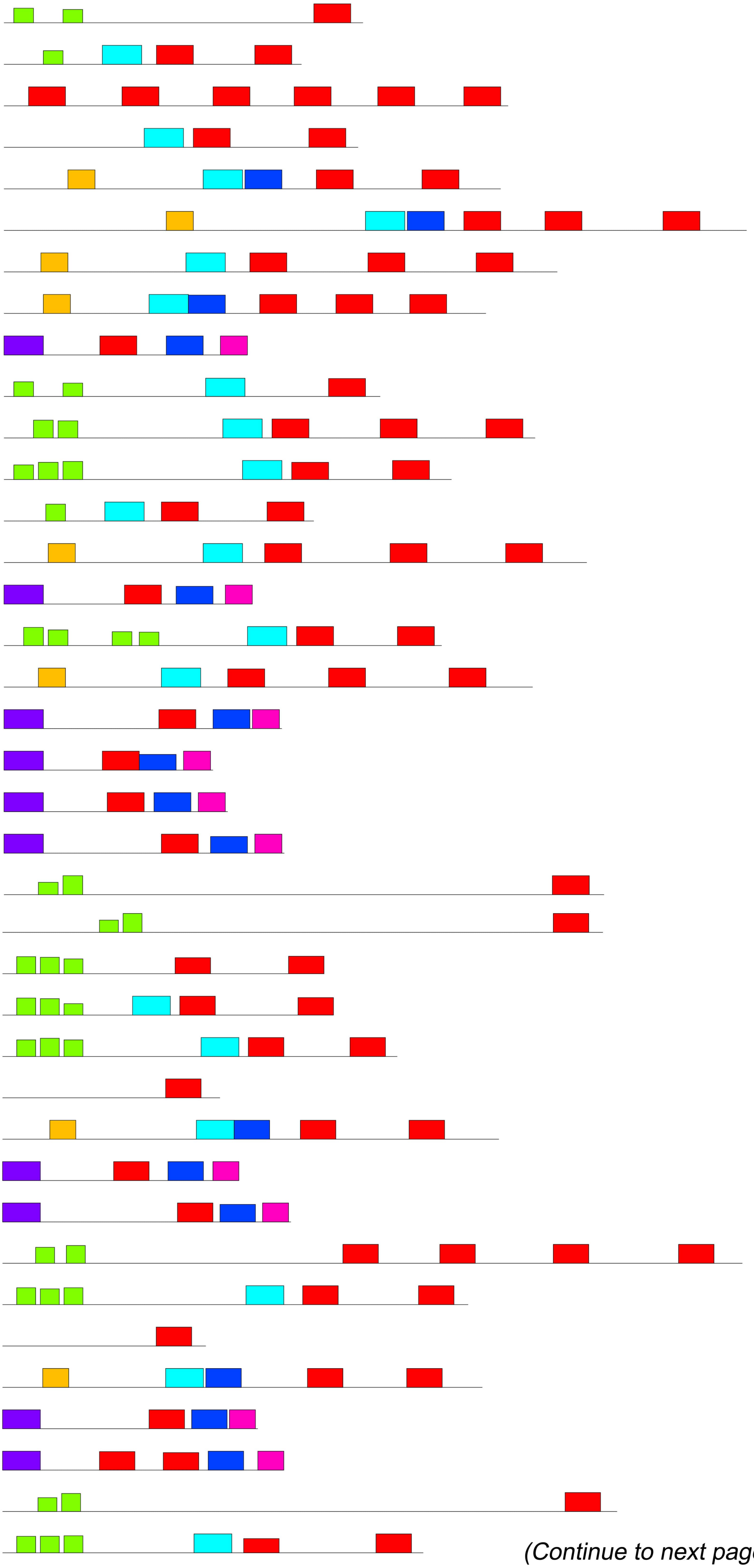

(Continue to next page)

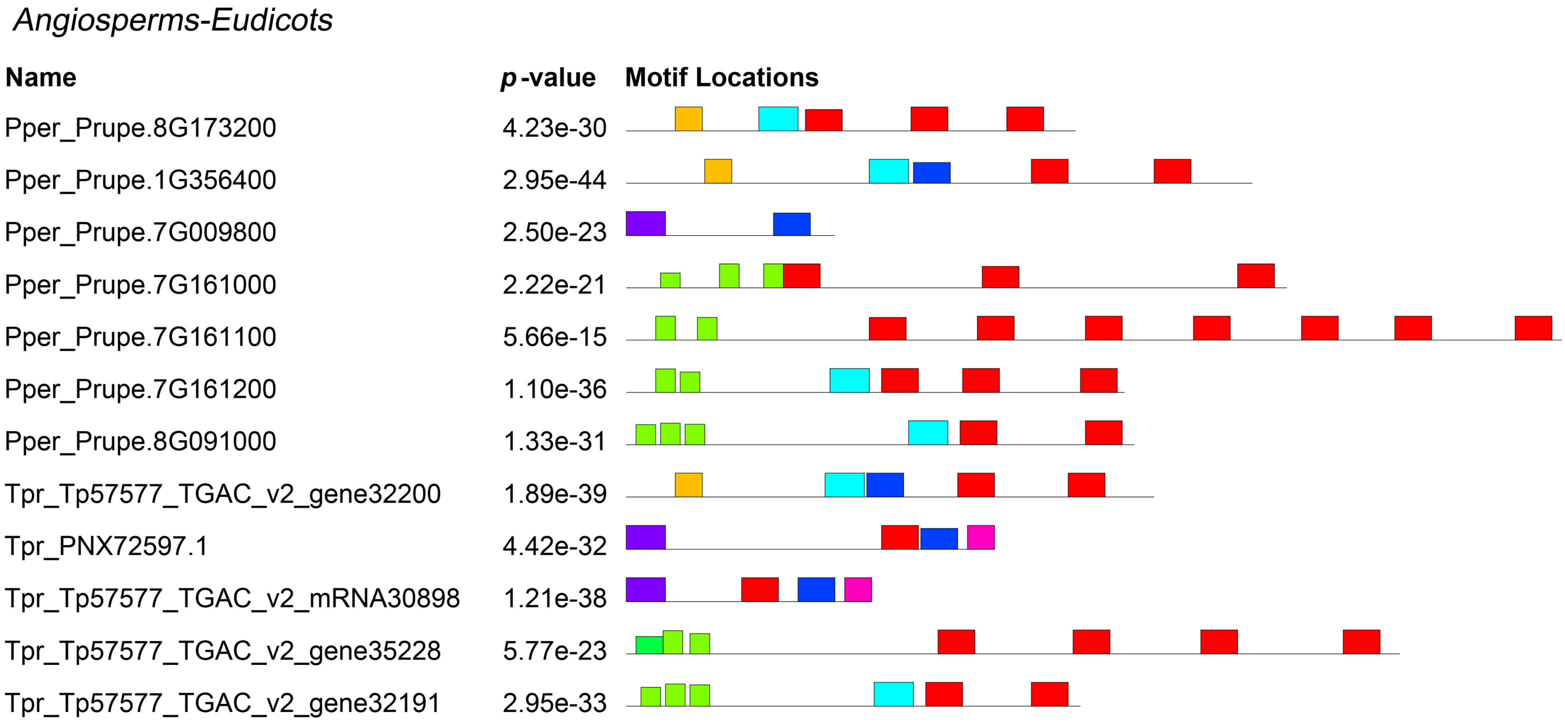

Motif	Symbol	Motif Consensus
1. *K-segment*		EKKGLMEKIKEKLPG
2. *S-segment*		LHRSGSSSSSSSEEEG
3. *Y-segment*		TDEYGNPV
4. *H-segment*		MAGIIHKIEETLHIGG
5. *F-segment*		EVKDRGLFDFL
6. *Phi-segment*		TQGGYGQQGHN
7. *B-segment*		DGEKKKKKKKKKKH
8. *S2-segment*		GGHSSSSSDSD

Figure S1. MEME analysis of the unbiased DHN database. *Each type of segment is indicated by a box with a different colour. For convenience, the sequences are shown ordered by taxonomic group.*

Supplementary Figures S2-S8 for Melgar and Zelada (2021)

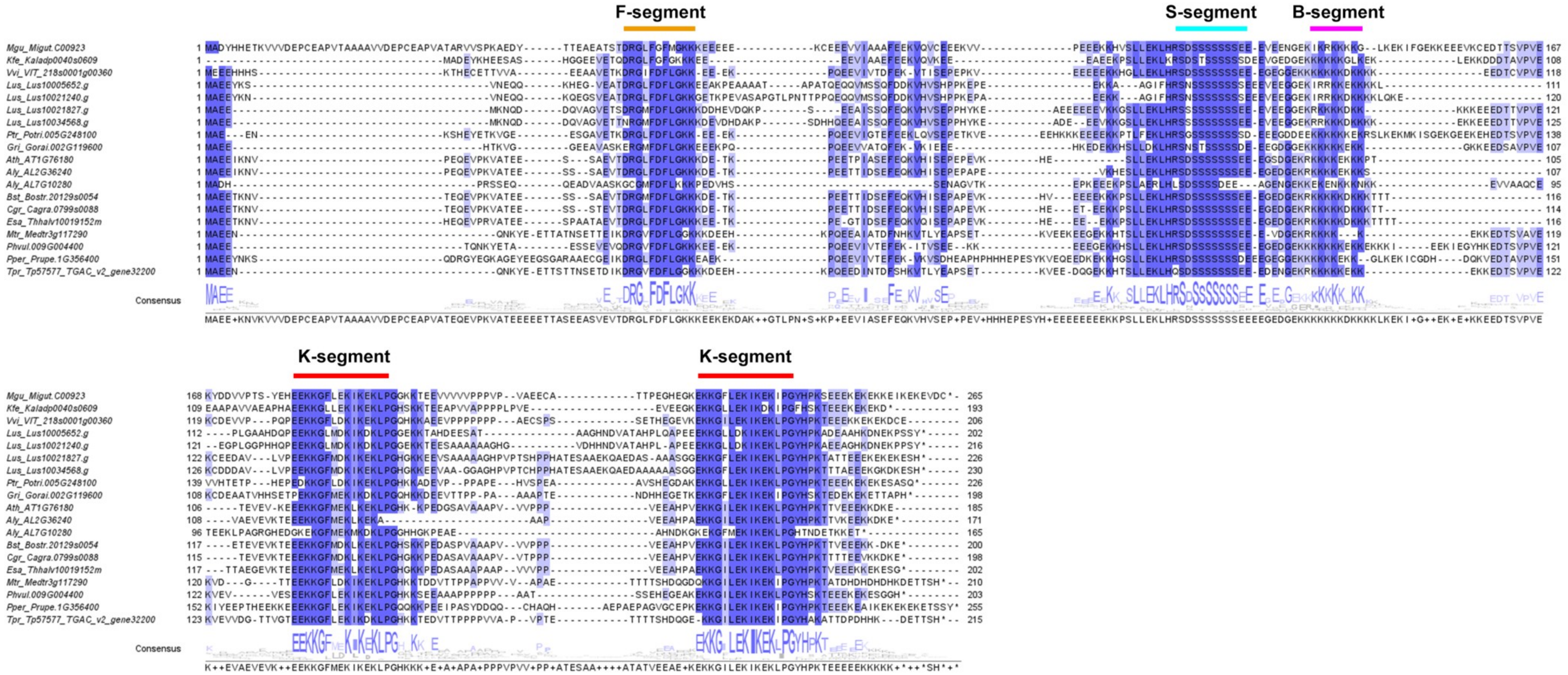

Figure S2. Multiple sequence alignment of FSK2 dehydrins. Protein sequences of FSK2 from eudicot species were aligned with Clustal Omega and visualised with Jalview. Structural segments are indicated and a consensus sequence is shown below the alignment.

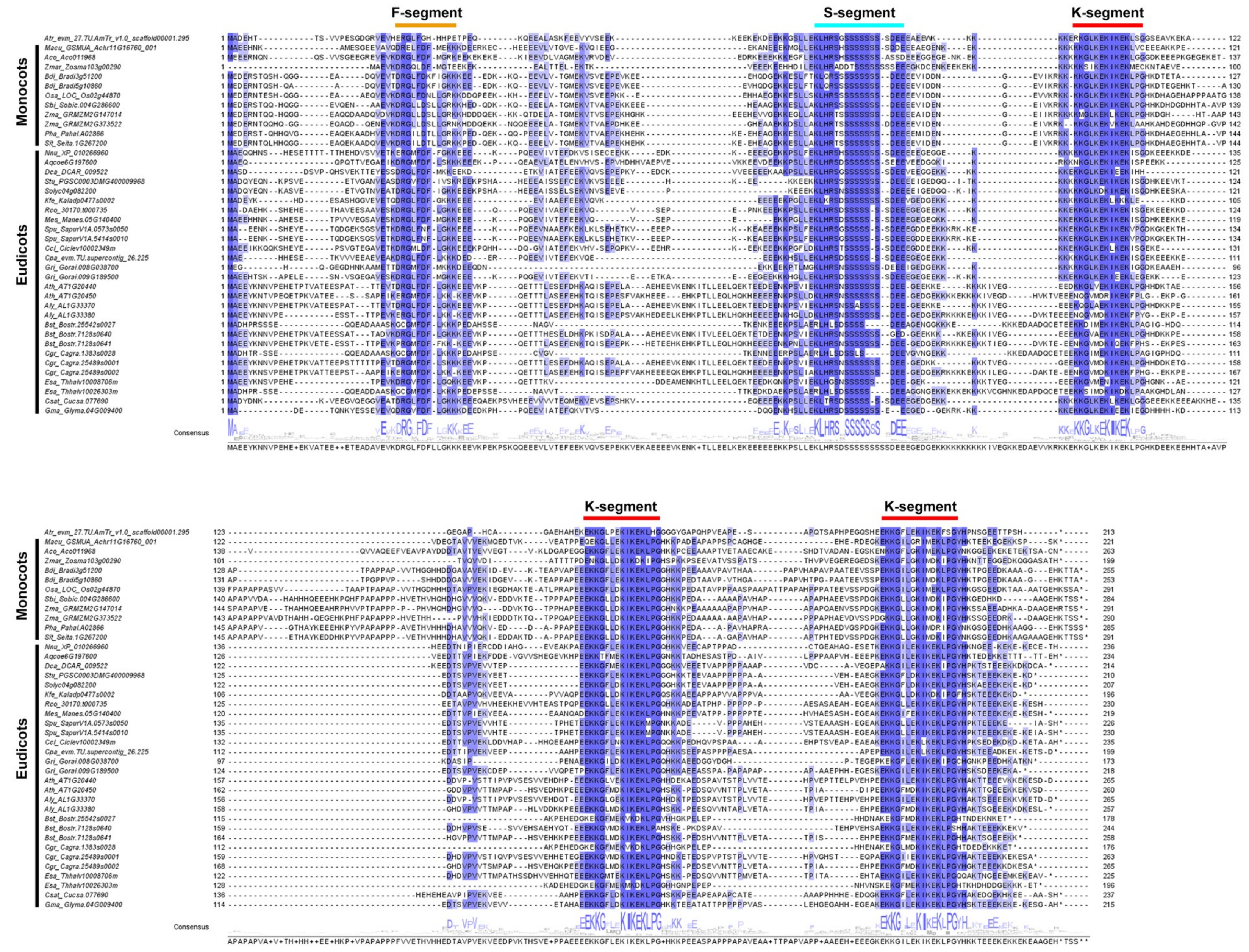

Figure S3. Multiple sequences alignment of FSK3 dehydrins. Protein sequences of FSK3-DHNs from angiosperms were aligned with Clustal Omega and visualised with Jalview. Structural segments are indicated and a consensus sequence is shown below the alignment. Note that there is a lysine-rich region adjacent to the S-segment but it is not as conserved as the B-segment found in FSK2-DHNs (compare to Fig. S2).

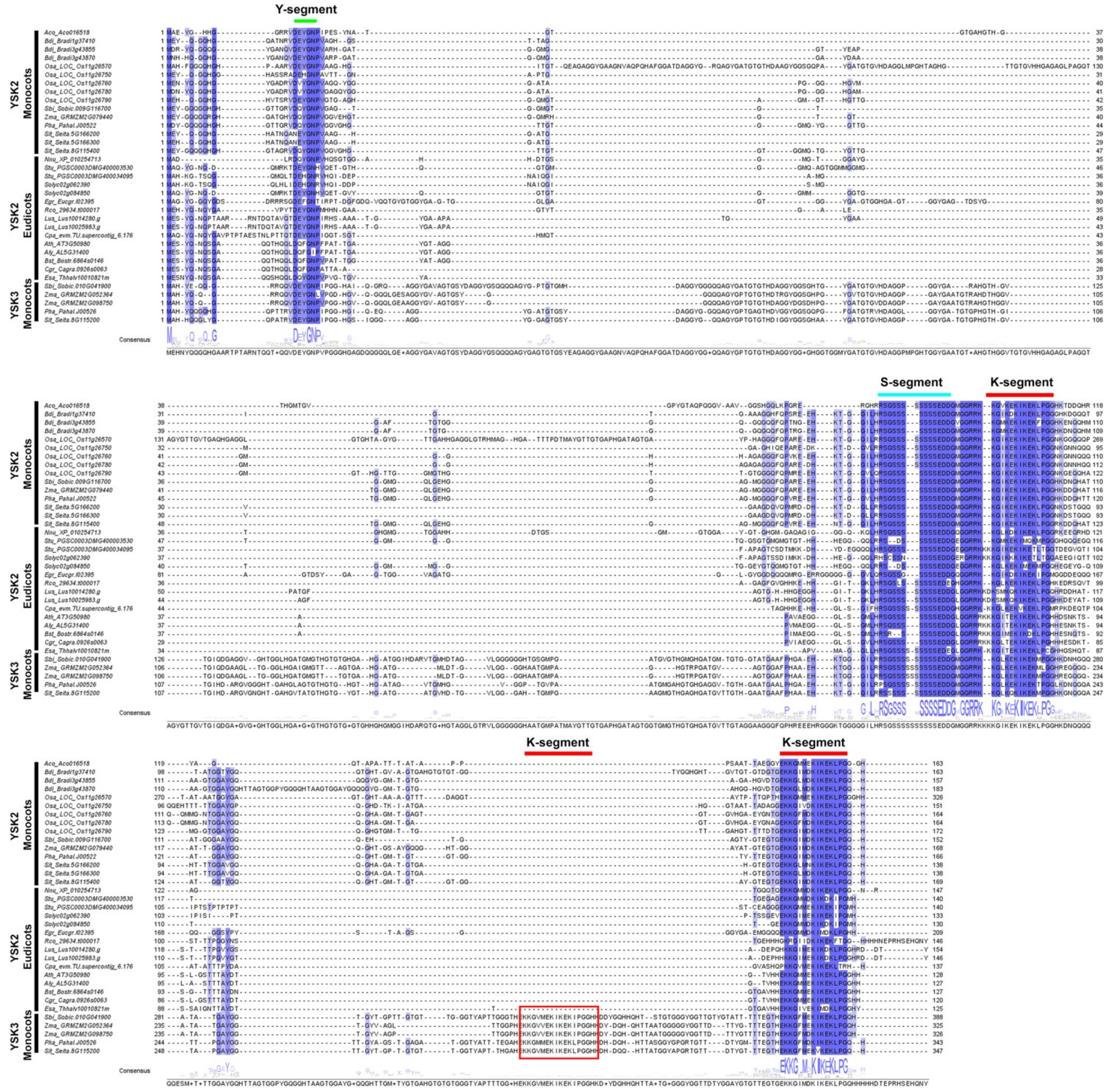

Figure S4. Multiple sequences alignment of YSKn dehydrins. Protein sequences of YSKn-DHNs from angiosperms were aligned with T-Coffee and visualised with Jalview. Structural segments are indicated and a consensus sequence is shown below the alignment.

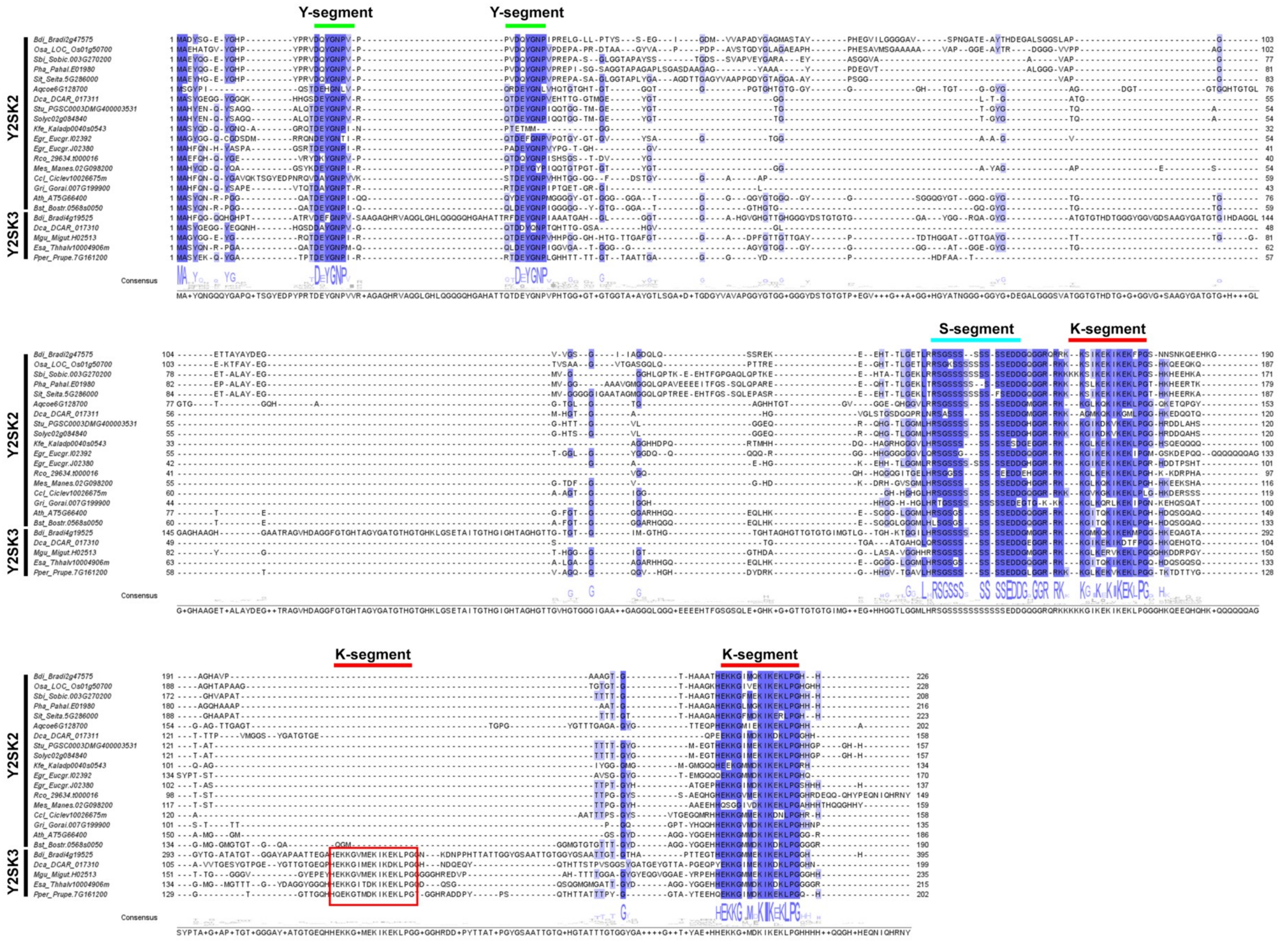

Figure S5. Multiple sequence alignment of dehydrins Y2SKn. Protein sequences of Y2SKn-DHNs from angiosperms were aligned with T-Coffee and visualised with Jalview. Structural segments are indicated and a consensus sequence is shown below the alignment. Y2SK2 and Y2SK3-DHNs are present in eudicots and the grass *Brachypodium distachyon*, while other Poaceae only have Y2SK2-DHNs.

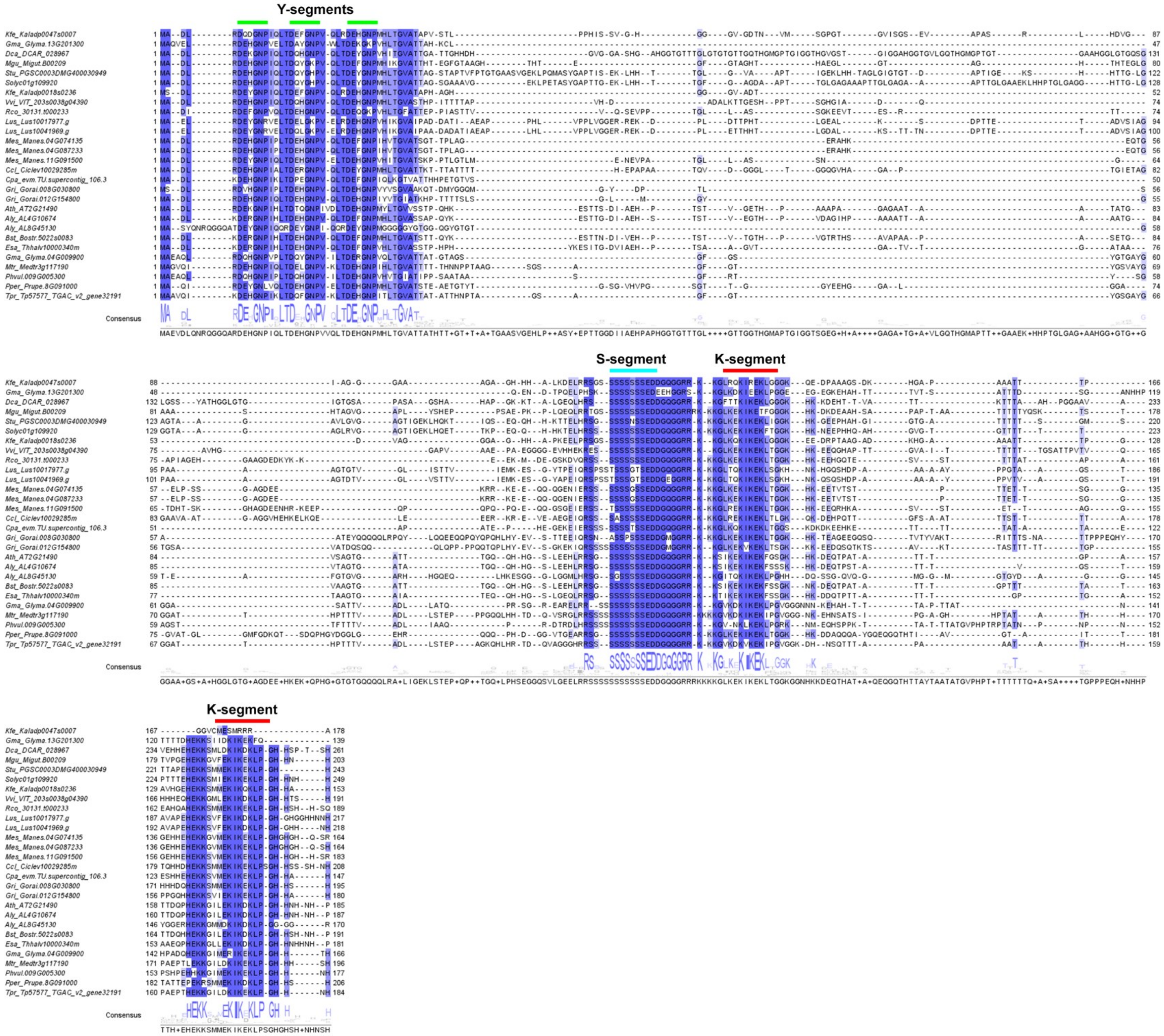

Figure S6. Multiple sequences alignment of Y3SKn dehydrins. Protein sequences of Y3SKn-DHNs from angiosperms were aligned with T-Coffee and visualised with Jalview. Structural segments are indicated and a consensus sequence is shown below the alignment.

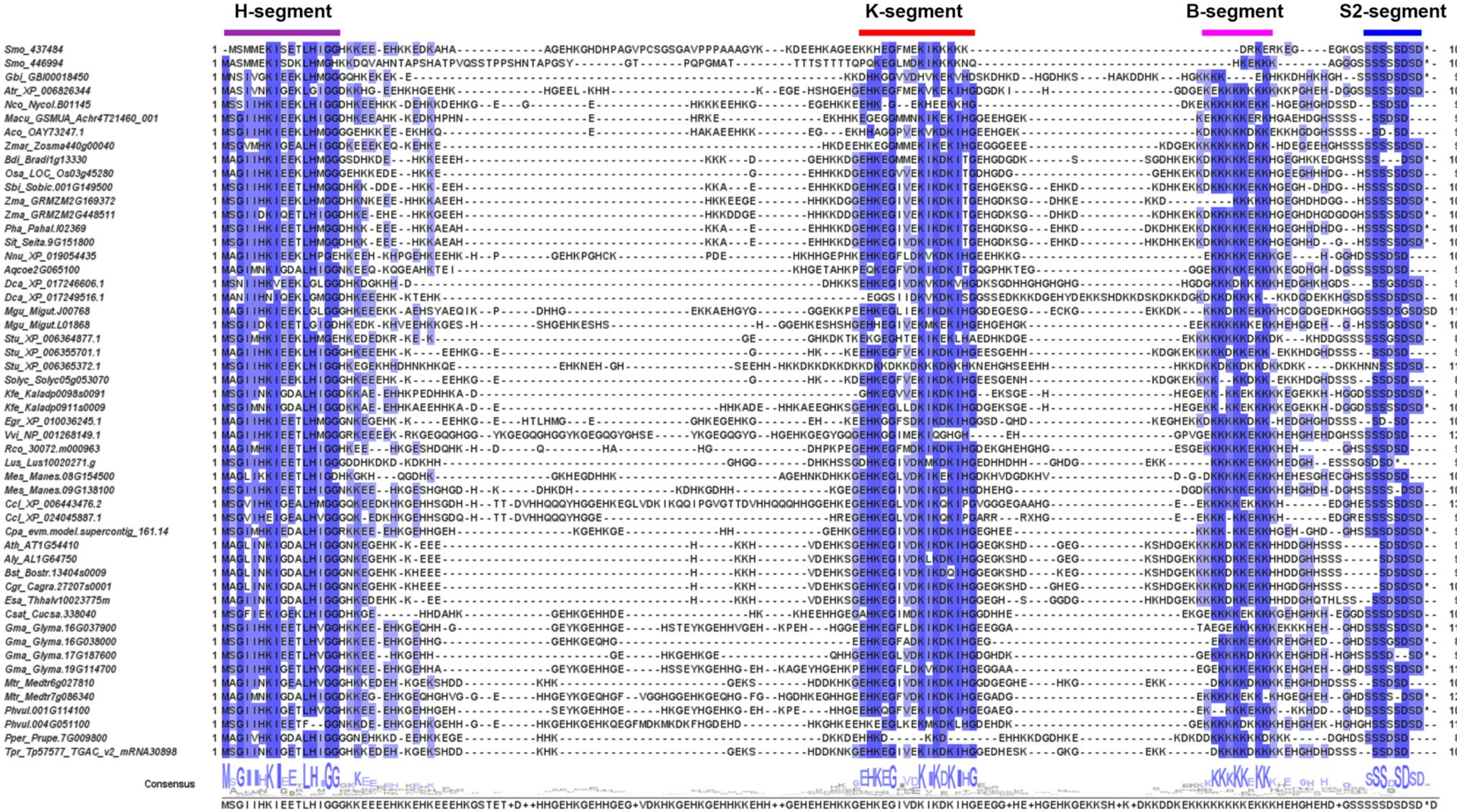

Figure S7. Multiple sequence alignment of HKS-dehydrins. Protein sequences of H-DHNs from vascular plants were aligned with Clustal Omega and visualised with Jalview. Structural segments are indicated and a consensus sequence is shown below the alignment. DHNs come from angiosperms except for proteins from *Selaginella moellendorffii* (Smo) and *Ginkgo biloba* (Gbi).

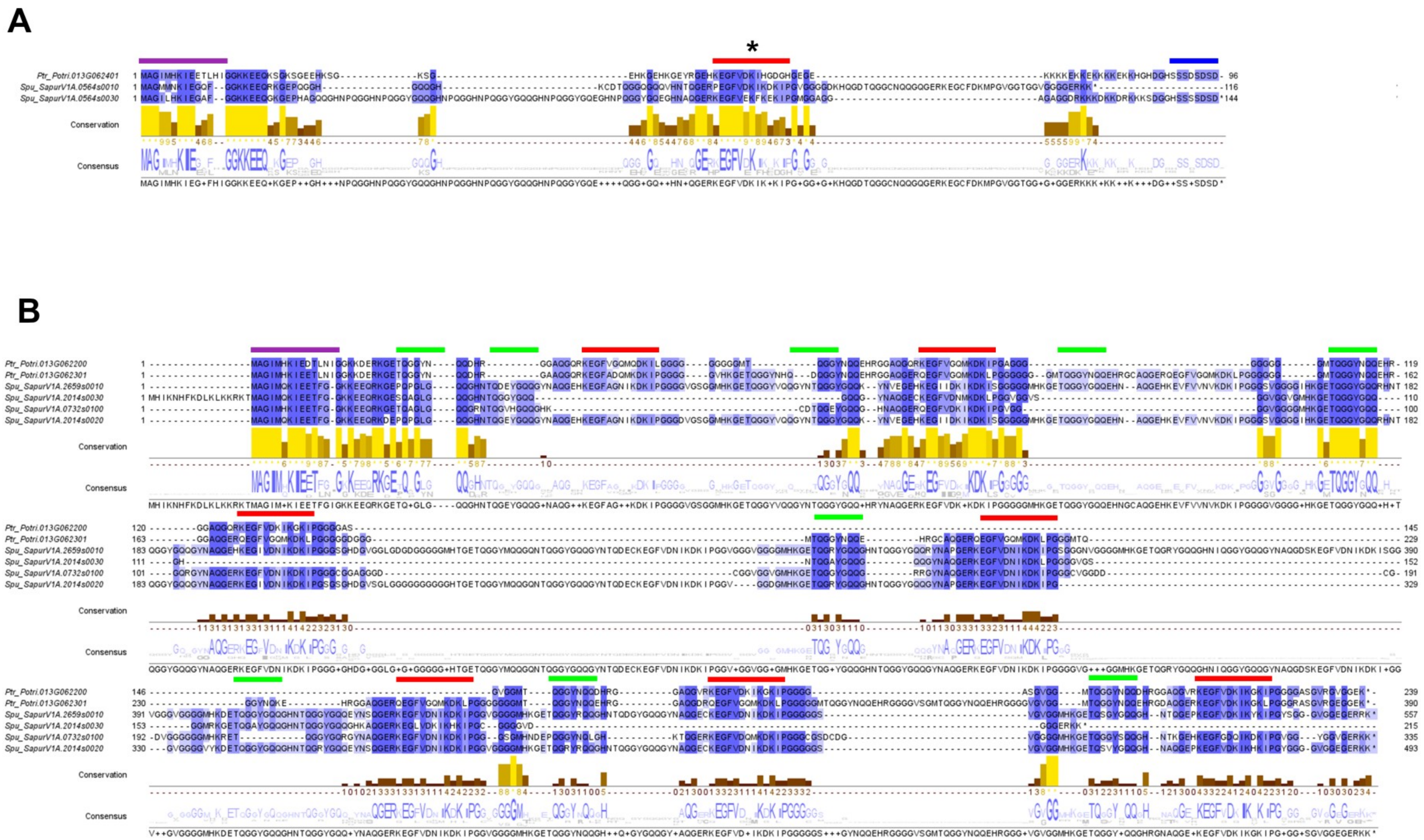

Figure S8. Multiple sequence alignment of atypical H-DHNs from Malpighiales. (**A**) Alignment of HKS-DHNs from *P. trichocarpa* and *S. purpurea* and a HS-DHN from *P. trichocarpa*. (**B**) Atypical H-DHNs with multiple K segments interspersed with Phi-segments. Segments are indicated by a colour code: H (purple), K (red), S2 (blue) and Phi (green). Sequences were aligned with Clustal Omega and visualised with Jalview.

A

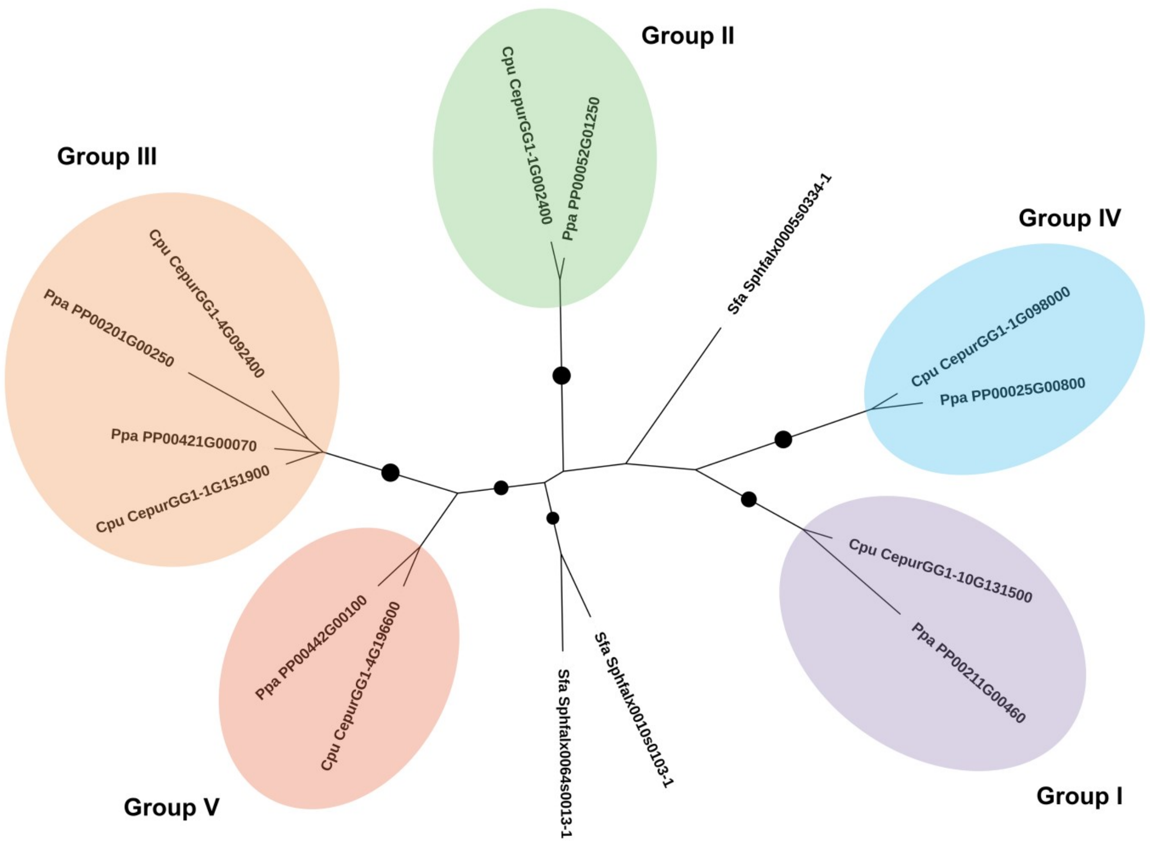

B

	DHN orthologous groups	
	Physcomitrella patens	***Ceratodon purpureus***
**Group I**	PP00211G00460 (PpDHNA)	CepurGG1.10G131500
**Group II**	PP00052G01250 (PpDHNB)	CepurGG1.1G002400
**Group III**	PP00421G00070 (PpDHNC)	CepurGG1.1G151900
	PP00201G00250 (PpDHND)	CepurGG1.4G092400
**Group IV**	PP00025G00800	CepurGG1.1G098000
**Group V**	PP00442G00100	CepurGG1.4G196600

Figure S9. Evolutionary relationships of bryophyte dehydrins. (**A**) Maximum-likelihood phylogenetic tree constructed with PhyML 3.0. Branches with bootstrap values over 90 are indicated with a circle. Note that DHN sequences from *P. patens* and *C. purpureus* form five homologous groups, while *S. fallax* DHNs are not grouped with the other sequences. (**B**) DHN sequences and homologous groups of *P. patens* and *C. purpureus*.

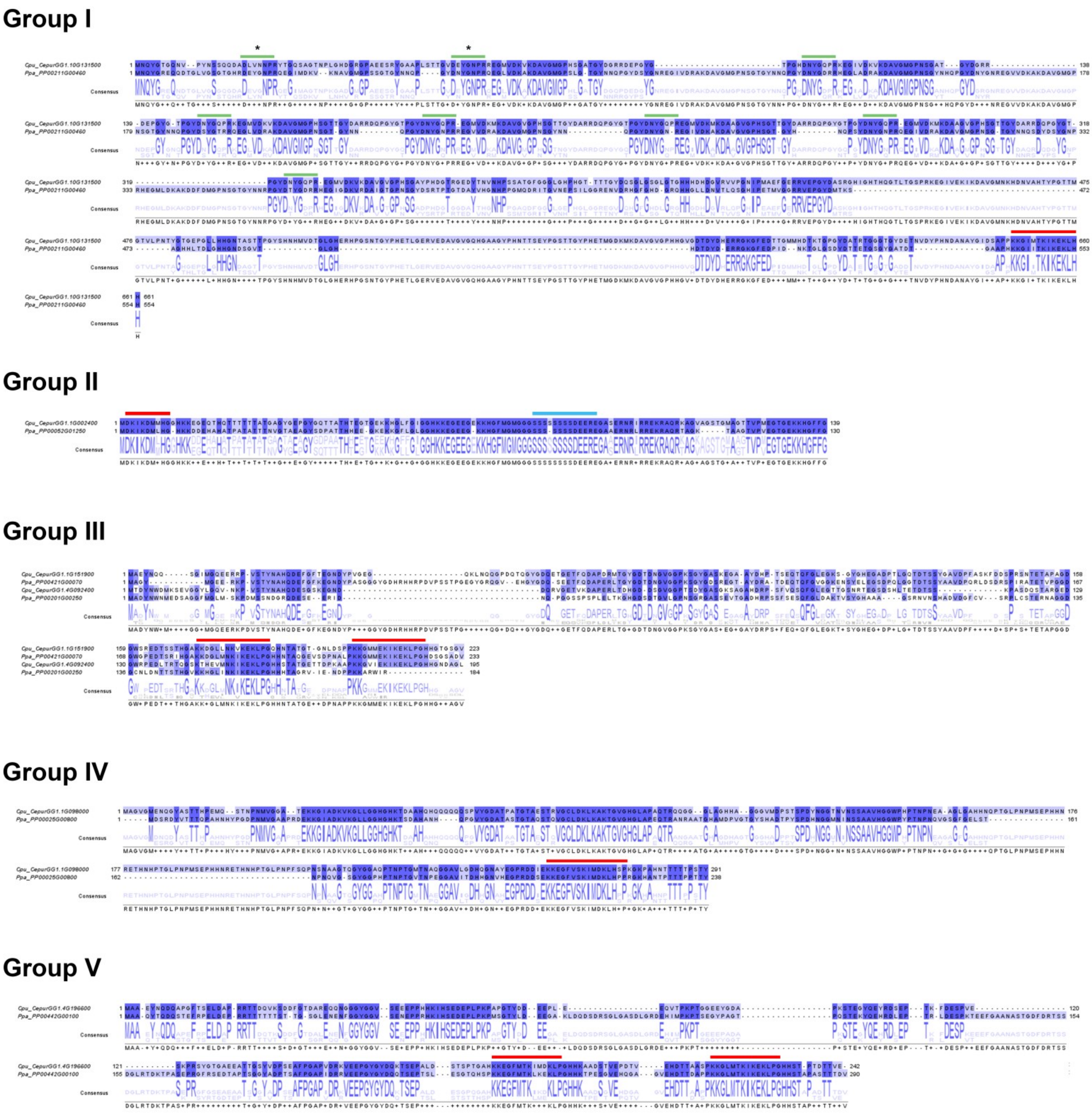

Figure S10. Multiple sequence aligment of bryophyte DHNs. Segments are indicated by a colour code: K (red), Y (green) and S (blue). Note that Group I has a Y8K structure; the Y-segments with an asterisk (*) have a sequence identical to the Y-segments of angiosperms (DEYGNP), while the others have a modified Y-segment (DNYGN/QP). Group II has a KS-structure, Group III and V have a K2-structure and Group IV a K-structure. Sequences were aligned with T-Coffee and visualised with Jalview.

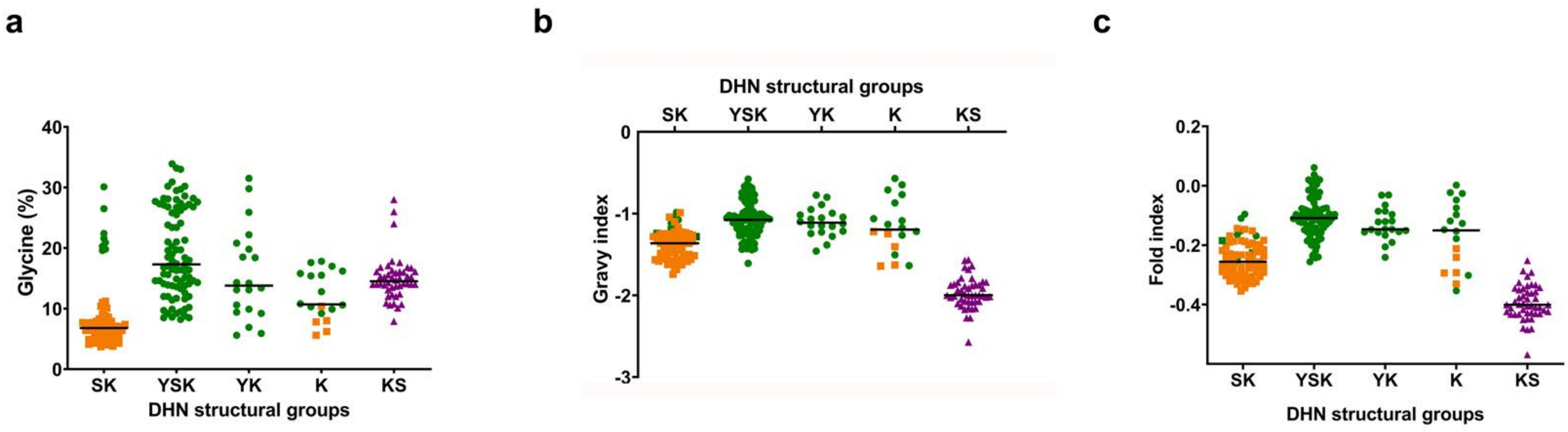

Figure S11. Scatter plots of physicochemical features of angiosperms DHN-structural groups: Glycine, GRAVY index and Fold index. Homologous groups are colour-coded: H-DHNs (purple) F-DHNs (orange) and Y-DHNs (green).
